# NEK10 interactome and depletion reveal new roles in mitochondria

**DOI:** 10.1186/s12953-020-00160-w

**Published:** 2020-04-28

**Authors:** Andressa Peres de Oliveira, Fernanda Luisa Basei, Priscila Ferreira Slepicka, Camila de Castro Ferezin, Talita D. Melo-Hanchuk, Edmarcia Elisa de Souza, Tanes I. Lima, Valquiria Tiago dos Santos, Davi Mendes, Leonardo Reis Silveira, Carlos Frederico Martins Menck, Jörg Kobarg

**Affiliations:** 1 Instituto de Biologia, Departamento de Bioquímica e Biologia Tecidual, Universidade Estadual de Campinas, Campinas, São Paulo, Brazil; 2 Departamento de Microbiologia, Instituto de Ciências Biomédicas, Universidade de São Paulo, São Paulo, Brazil; 3 Faculdade de Ciências Farmacêuticas, Universidade Estadual de Campinas, Rua Cândido Portinari, 200; Cidade Universitária Zeferino Vaz; Campinas-SP; CEP, São Paulo, 13083-871 Brazil; 4 Laboratório Nacional de Biociências, Centro Nacional de Pesquisa em Energia e Materiais, Campinas, São Paulo, Brazil; 5 Departamento de Biologia Estrutural e Funcional, Instituto de Biologia, Universidade Estadual de Campinas, Campinas, São Paulo, Brazil; 6 Departamento de Bioquímica e Imunologia, Universidade de São Paulo, Ribeirão Preto, Brazil

**Keywords:** NEK10, Interactomics, Mitochondrial dynamics and metabolism, mtDNA

## Abstract

**Background:**

Members of the family of NEK protein kinases (NIMA-related kinases) were described to have crucial roles in regulating different aspects of the cell cycle. NEK10 was reported to take part in the maintenance of the G2/M checkpoint after exposure to ultraviolet light. NEK1, NEK5, NEK2 and NEK4 proteins on the other hand have been linked to mitochondrial functions.

**Methods:**

HEK293T cells were transfected with FLAG empty vector or FLAG-NEK10 and treated or not with Zeocin. For proteomic analysis, proteins co-precipitated with the FLAG constructs were digested by trypsin, and then analyzed via LC-MS/MS. Proteomic data retrieved were next submitted to Integrated Interactome System analysis and differentially expressed proteins were attributed to Gene Ontology biological processes and assembled in protein networks by Cytoscape. For functional, cellular and molecular analyses two stable Nek10 silenced HeLa cell clones were established.

**Results:**

Here, we discovered the following possible new NEK10 protein interactors, related to mitochondrial functions: SIRT3, ATAD3A, ATAD3B, and OAT. After zeocin treatment, the spectrum of mitochondrial interactors increased by the proteins: FKBP4, TXN, PFDN2, ATAD3B, MRPL12, ATP5J, DUT, YWHAE, CS, SIRT3, HSPA9, PDHB, GLUD1, DDX3X, and APEX1. We confirmed the interaction of NEK10 and GLUD1 by proximity ligation assay and confocal microscopy. Furthermore, we demonstrated that NEK10-depleted cells showed more fragmented mitochondria compared to the control cells. The knock down of NEK10 resulted further in changes in mitochondrial reactive oxygen species (ROS) levels, decreased citrate synthase activity, and culminated in inhibition of mitochondrial respiration, affecting particularly ATP-linked oxygen consumption rate and spare capacity. NEK10 depletion also decreased the ratio of mtDNA amplification, possibly due to DNA damage. However, the total mtDNA content increased, suggesting that NEK10 may be involved in the control of mtDNA content.

**Conclusions:**

Taken together these data place NEK10 as a novel regulatory player in mitochondrial homeostasis and energy metabolism.

## Background

Protein kinases comprise 1.7% of all genes in the human genome and participate in biological processes that are crucial and evolutionary conserved in eukaryotes [1]. Initially, the protein NIMA (Never in mitosis gene A) was identified in the fungus *Aspergillus nidulans* as a serine/threonine kinase (79 kDa), crucial for the mitotic entry. However, studies indicated that NIMA has roles in all phases of cell division [2–4].

In mammals, eleven protein kinases share 40–50% amino acid sequence identity in their catalytic domain with that of NIMA, and were hence denominated as NEKs: NIMA related kinases.

NEK10 structure is unique in that it has a central catalytic kinase domain, flanked by two large regulatory domains. Like NIMA and NEKs 1, 2, 5, 9, and 11, the NEK10 protein has a coiled-coil region, located near the kinase domain. In the amino-terminal regulatory domain there are four armadillo motifs, which also act as key regions for protein-protein interactions [5].

Mutations in NEK10 have been reported in lung cancer [6] and breast cancer, in which polymorphisms in BRCA1/2 (breast cancer type 1/2 susceptibility protein) were found [1, 7]. Moniz and Stambolic [8] reported a role of NEK10 protein in the maintenance of the G2/M checkpoint, followed by ultraviolet (UV) irradiation. The NEK10 protein acts as a positive regulator of ERK1/2 (Extracellular signal-regulated protein kinases 1 and 2), after UV irradiation and forms a complex with RAF1 and MEK1.

Recently, a report showed that NEK10 is important for ciliogenesis. NEK10 interacts with PKA and PCM1 and participates in a cAMP dependent pathway, contributing to cilium formation [9]. Mitochondria are cytosolic organelles, with double membranes and their own genomes [10]. They are involved in energy production, Ca^2+^ homeostasis, cell death, chronic inflammation and the aging process [11]. Changes in mitochondrial homeostasis contribute to metabolic disorders, cardiomyopathies, neurodegeneration and cancer [11].

Recently, some NEK proteins have been linked to mitochondrial functions. NEK1 regulates cells death through phosphorylation of voltage dependent anion channel 1 (VDAC1) on serine 193 [12, 13]. Cells silenced for NEK5 showed increased levels of reactive oxygen species (ROS) formation and cell death, probably mediated through deficient regulation in the complex IV of the respiratory chain [14]. Also, NEK2 has an important role in aerobic glycolysis by regulating the splicing of PKM and increasing the PKM2/PKM1 ratio in myeloma cells [15]. NEK4 also interacted with several mitochondrial proteins and ongoing functional assays promise to provide interesting new insights [16].

Through Mass Spectrometry (MS) analyses of immunoprecipitated (IP) samples, we identified mitochondrial proteins as NEK10 interactors. Among them were Glutamate dehydrogenase (GLUD1) and Citrate Synthase (CS). This prompted us to investigate the role of NEK10 in mitochondrial morphology, respiration, ROS production, citrate synthase activity, mtDNA integrity and mtDNA copy numbers. Together, our data add NEK10 as another protein of the NEK family to be involved in mitochondrial functions and thereby seem to point to the NEKs as kinases that regulate the functional crosstalk of cell cycle checkpoints with mitochondria [17, 18].

## Methods

### Cell culture

HEK293T, HeLa and MRC5 human cell lines were obtained from ATCC. Cells were maintained in a humid incubator with 5% CO_2_ at 37 °C and cultivated in high glucose Dulbecco’s modified Eagle’s medium (Gibco Thermo Fisher Scientific, Waltham, MA, USA) enriched with 10% certified fetal bovine serum (Gibco) and penicillin/streptomycin (100 units/mL, Gibco). The Zeocin antibiotic (Invitrogen, Thermo Fisher Scientific) was purchased ready to use.

### Knock down of NEK10 in HeLa cells using short hairpin RNA

We used a lentiviral short-interfering RNAs (shRNAs) system to target NEK10 (shNEK89: 5′-CATTGCCAGAACACATTATAT-3′; shNEK90: 5′-GCTCGTCCAGATATTGTAGAA-3′). pLKO.1 empty vector was used as control (pLKO.1) and the shRNAs were obtained from The RNAi Consortium (TRC, IRB-Barcelona, Spain). Lentiviruses carrying shRNAs were produced and harvested at The Viral Vector Laboratory (LVV, LNBio/CNPEM-Campinas, SP, Brazil). Lentiviruses were transduced in HeLa cells in the presence of 1 μg/mL polybrene and complete medium for 24 h. Stable HeLa NEK10-depleted cell lines. For selection we used puromycin (Sigma-Aldrich, St Louis, MO, USA) at 3 μg/mL and regularly tested for mycoplasma contamination.

### Antibodies and fluorescent dyes

Primary antibodies used: rabbit anti-NEK10 (Atlas Antibodies® HPA, Cat# 038941 1/250); mouse anti-OXPHOS (Abcam Rodent WB Antibody Cocktail, Cat# ab110413–1/500); mouse anti-GAPDH (Millipore, Cat# MAB374–1:500); mouse anti-Lamin A/C (Santa Cruz Biotechnology, Cat# sc-7292 - 1:200); goat anti-TFAM (Santa Cruz Biotechnology E-16, Cat# sc-30963 - 1:100); rabbit anti-VDAC (Cell Signaling Technology, Cat# 4866, 1:1000); rabbit anti-Tubulin B (Abcam, Cat# ab15568 1:1000).

The specificity of mouse anti-NEK10 antibody was performed using immunofluorescence assays. HeLa cells transfected with FLAG-NEK10 presented an increase in fluorescence after staining with mouse NEK10 antibody. The plasmid pcDNA6-FLAG-Ki-1/57 [19] was used as a negative control (Supporting Figure S4). The goat anti-NEK10 antibody was used as previously described [9]. The rabbit anti-NEK10 datasheet shows the specificity for the 80 kDa NEK10 isoform (Atlas Antibodies).

For immunoprecipitation: mouse anti-NEK10 (Santa Cruz Biotechnology, Cat# sc-100434 -1/100); normal mouse IgG (Santa Cruz Biotechnology, Cat# sc-2025, 1:1000); mouse anti-FLAG M2 (Sigma-Aldrich, Cat# A2220).

For immunofluorescence: goat anti-GLUD1 (US Biological, Cat# G4000-51C - 1:100); goat anti-NEK10 (Santa Cruz Biotechnology, Cat# sc-103067 and sc-103067 - 1:100), mouse anti-NEK10 (Santa Cruz Biotechnology, Cat# sc-100434, 1:25 or 1:100 for validation experiments); Alexa Fluor conjugated secondary antibodies were used at 1:300 dilution: Anti-Goat Alexa Fluor 647; Anti-mouse Alexa Fluor 488; Anti-Goat Alexa Fluor 488.

Mitotracker™ Deep Red FM (Invitrogen, Cat# M22426) MitoSOX™ (Invitrogen, Cat# M36008).

For proximity ligation assay**:** goat anti-GLUD1 (US Biological, Cat# G4000-51C - 1:50); goat anti-NEK10 (Santa Cruz Biotechnology, Cat# sc-103067 - 1:50), mouse anti-NEK10 (Santa Cruz Biotechnology, Cat# sc-100434 1:25) and mouse anti-citrate synthase (Santa Cruz Biotechnology G-3, Cat# sc-390693 -1:25).

### Immunoprecipitation followed by mass spectrometry (IP-LC-MS/MS)

HEK293T cells were transfected with FLAG empty vector or FLAG-NEK10 for 48 h and a subset of both pools of cells was treated with 300 μg/mL Zeocin for 3 h. Cells were lysed with lysis buffer (50 mM Tris 7,4, 100 mM NaCl, 1 mM DTT, 1 mM EDTA, 30 μg/mL DNase I, 1% Triton X- 100) supplemented with protease inhibitor cocktail (Roche Applied Science, Mannheim, Germany). FLAG and FLAG-NEK10 lysates were incubated with anti-FLAG M2 Agarose Affinity Gel (Sigma- Aldrich) during 16 h at 4 °C. Immunoprecipitated (IP) complexes using anti-FLAG were eluted with 3x FLAG peptide and lysates and IP samples were resolved on SDS-PAGE gels. For visualization of FLAG-NEK10 expression and immunoprecipitation quality, SDS-PAGE gels were immunoblotted using anti-FLAG antibody and silver stained, as described [20].

For proteomic analysis, the immune-complexes were reduced in 5 mM dithiothreitol for 30 min at 56 °C, alkylated with 14 mM iodoacetamide for 30 min at room temperature, protected from the light, and digested with 20 ng/μl trypsin (Promega, Madison, WI, USA). The digested peptides were dried in a vacuum concentrator, reconstituted in 30 μl of 0.1% formic acid. An aliquot of 4.5 μL was analyzed on an ETD enabled Orbitrap Velos mass spectrometer (Thermo Fisher Scientific, Waltham, MA, USA) connected to the EASY-nano Liquid Chromatography (LC-MS/MS system (Proxeon Biosystem, West Palm Beach, FL, USA) through a Proxeon nano-electrospray ion source as previously described [21]. Peptides were separated by a 2–30% acetonitrile gradient in 0.1% formic acid using an analytical column PicoFrit Column (20 cm x ID75 μm, 5 μm particle size, New Objective) at a flow rate of 300 nL·min^− 1^ over 30 min. The nano-electrospray voltage was set to 2.2 kV and the source temperature was 275 °C. All instrument methods were set up to the data dependent acquisition mode. The full scan MS spectra (m/z 300–1600) were acquired on the Orbitrap analyzer after accumulation to a target value of 1 × 10^6^. The resolution in the Orbitrap was set to r = 60,000 and the 20 most intense peptide ions with charge states ≥2 were sequentially isolated to a target value of 5000 and fragmented in the linear ion trap using low-energy CID (normalized collision energy of 35%). The signal threshold for triggering an MS/MS event was set to 1000 counts. Dynamic exclusion was enabled with an exclusion size list of 500, exclusion duration of 60 s, and a repeat count of 1. An activation q = 0.25 and an activation time of 10 ms were used.

Peak lists (msf) were generated from the raw data files using Proteome Discoverer version 1.4 (Thermo Fisher Scientific) with Sequest search engine and searched against UniProt Human Protein Database (released on January 22nd; 2014; 88,429 sequences, 35,079,223 residues) with carbamidomethylation (+ 57.021 Da) as fixed modification, oxidation of methionine (+ 15.995 Da), as variable modification, one trypsin missed cleavage and a tolerance of 10 ppm for precursor and 1 Da for fragment ions, using the FDR less than 1%.

The peptides identified in the mass spectrometry were considered only if: (1) immunoprecipitated in at least 2 replicates of FLAG-NEK10 samples and (2) detected in none or only one replicate of control-FLAG immunoprecipitates. The LC-MS/MS analyses were performed at the Mass Spectrometry Facility in the Brazilian Bioscience National Laboratory (LNBio-CNPEM, Campinas-SP-Brazil).

### In silico protein-protein interaction network analysis

The proteomic data retrieved from IP-LC-MS/MS was submitted to the Integrated Interactome System [22]. The Gene Ontology (GO) biological processes were obtained by using hypergeometric distribution, as described previously [23] and the sub-cellular localization (cellular component –CC) was defined according to the selected CC obtained from the annotation and interactome module. Cytoscape 3.7.0 [24] was used to assemble the protein networks.

### Isolation of crude mitochondria

HeLa pLKO, HeLa pLKO-sh89, HeLa pLKO-sh90, MRC5 and HEK293T cells were rinsed with 5 ml of PBS (Phosphate buffered saline), trypsinized and centrifuged at 250 x *g* for 5 min*.* Cell pellets were resuspended in 4.5 mL of 1x IB-1 buffer (225 mM mannitol, 75 mM sucrose, 0.1 mM EGTA, 30 mM TRIS-HCl pH 7.4) and homogenized by approximately 100 strokes in a glass-homogenizer, manually. The homogenate (whole cell extract) was centrifuged for 10 min at 800×*g* and the pellet discarded, twice. Approximately 100 μl of the postnuclear supernatant (PNS) were harvested for immunoblot analyses. The remaining PNS was centrifuged at 10.000 x *g* for 20 min, and the resulting pellet (MITO) and cytosolic fraction (CYT) were separated for two different procedures. The MITO fraction was washed with ice-cold IB-1 buffer and centrifuged at 8.500 x *g* for 10 min. The pellet was resuspended in 1x IB-2 buffer (225 mM mannitol, 75 mM sucrose, 30 mM TRIS-HCl pH 7.4), followed by centrifugation at 10.000×*g* for 10 min, twice. After centrifugation, the pellet was resuspended in 800 μl of MRB (Mitochondrial resuspending buffer = 250 mM mannitol, 5 mM HEPES pH 7.2, 0.5 mM EGTA). The 1 ml of cytosolic fraction (CYT) was ultra-centrifuged at 34.000 rpm for 1 h at 4 °C (Optima L-90 K Beckman-Coulter Rotor: sw -41Ti speed: 28.500 rpm). This protocol was adapted from a procedure previously described [25].

### Immunofluorescence and confocal assays

For NEK10 co-localization experiments, HeLa cells were dyed (MitoTracker Deep Red,200 nM)) during 20 min. After fixation with methanol, blocking occurred in PBS, containing 0,1% Triton X-100 and 3% of BSA. Then, incubation with primary and secondary antibodies occurred and both antibodies were diluted in the same buffer and incubated for 1 h and 20 min, respectively. Next, the coverslips were rinsed with PBS and prepared with ProLong (ThermoFisher) antifade reagent. The confocal acquisitions were carried out with confocal LSM 510 microscope and treated using FIJI software [26], at the INFABIC/UNICAMP (see Acknowledgments).

For colocalization analyses, images were processed with background correction using 50.0 pixels in ball rolling radius and applying median filtering. Fiji Coloc2 plugin was used for Pearsons’s correlation coefficient calculation [26]. Pearson’s correlation coefficient was calculated from the mean of at least 15 cells. For mitochondrial morphology analyses, HeLa pLKO-empty and HeLa Nek10 depleted (pLKO-sh89 and pLKO-sh90) cells were grown in culture dishes with glass bottom. Cells were stained with MitoTracker Deep Red (200 nM) during 20 min and then cell culture media was changed to medium without phenol red and immediately visualized under confocal LSM 510 microscope. Images were acquired using 63× oil objective, 1 air unit pinhole, 1024 × 1024 pixels. Scale bars: 10 μm. For morphology analyses, cells were classified on the basis of their filaments size and format: oval/spheres, short (< 6 μm) or long (> 6 μm) mitochondrial filaments as described before [27]. At least 30 cells per sample were counted.

### Proximity ligation assay

HeLa pLKO cells were plated in a 384 well cell carrier plate (PerkinElmer Inc) and the analyses were performed in triplicated. After 24 h, the cells were fixed with methanol and incubated in blocking solution (1X PBS, 0.1% Triton X-100, and 3% BSA). Cells were incubated with primary antibodies followed by anti-mouse plus and anti-goat minus probes and submitted to the further steps of the proximity ligation assay (PLA) kit’s protocol according to the manufacturer’s instructions. Nuclei were counter-stained with Hoechst 33342 dye. Red spots corresponding to NEK10 and GLUD1 or NEK10 and Citrate Synthase co-localizations per cell were visualized at ImageXpress Micro Imaging System and counted in MetaXpress software (Molecular Devices, San Jose, CA), as described previously [28]. The negative control samples were performed with the rolling-circle amplification reaction but without the primary antibodies or the probes.

### Immunoblot

Whole-cell lysates, quantified by Bradford’s Method [29], and immunoprecipitates were resolved by SDS-PAGE and blotted onto nitrocellulose membranes (Amersham, GE Healthcare Life Sciences, Pittsburgh, PA, USA). Then, the membranes were incubated with primary antibodies diluted in T-TBS buffer (Tris-buffered Saline containing 0,02% of Sodium Azide, 2% of bovine serum albumin and 0,5% of Tween 20) overnight at 4 °C. The secondary antibodies were left for 1 h at room temperature and after that the protein detection was performed using the enhanced chemiluminescence ECL Western Blotting System (Amersham).

### Relative mitochondrial DNA copy number

Total cellular DNA was isolated using the DNAeasy Mini Kit (Qiagen, Hilden, Germany), and DNA concentrations were determined spectrophotometrically with Nanodrop. Equal amounts of total DNA were assayed by quantitative PCR (qPCR) with SYBR Green mix (Applied Biosystems, Thermo Fisher Scientific). Samples were analyzed at least in triplicates, using two different DNA isolates, independently, in a 7300 Fast Real Time PCR System (Applied Biosystems, Thermo Fisher Scientific). The comparative Ct method was applied for quantification of mitochondria DNA copy number, comparing the amplification of ND1 (mitochondrial gene) to that of HPRT (nuclear gene). Primer pair sequences are as follows: ND1-F 5’ACT ACG CAA AGG CCC CAA CG 3`; ND1-R 5’GAG CTA AGG TCG GGG CGG TG 3`; HPRT1-F 5’TGA CAT GTG CCG CCT GCG AG 3`; HPRT1-R 5’GTG GGTC GCT TTC CGT GCC GA 3`.

### Oxygen consumption

HeLa pLKO, HeLa shNEK10–89 and HeLa shNEK10–90 cells were kept in Petri dishes, trypsinized, centrifuged at 1.500 rpm for 5 min and resuspended in DMEM medium. Cellular oxygen consumption (for 4 × 10^6^ cells) was employed in the high-resolution oxygraph chamber (Oroboros Oxygraph-2 K). Cells were incubated in a closed chamber containing 2 mL of DMEM medium (25 mM glucose, 20 mM Hepes, pH 7.4). After the initial reading, the Oroboros chamber was closed and baseline oxygen consumption (oxygen flow) was monitored, followed by addition of oligomycin (1 μg/mL) to obtain the consumption coupled to oxidative phosphorylation. In order to induce maximum mitochondrial respiration and to obtain mitochondrial reserve capacity (=spare capacity), 0.5 μM of Carbonyl cyanide m-chlorophenyl hydrazone (CCCP) titration was used. Then, 1 μM of Rotenone was added for mitochondrial complex I inhibition and to obtain non-mitochondrial oxygen consumption. Spare capacity was measured, subtracting basal respiration from maximal respiration capacity. The data were obtained through software DatLab 4 (Oroborus Instruments, Innsbruck, Austria).

### Flow cytometry

For mitochondrial ROS detection, MitoSOX™ Red (ThermoFisher scientific, Cat# M36008), specific for mitochondrial ROS was utilized. One day prior to the treatment, HeLa control or NEK10 depleted cells were plated in a 24 well plate. To visualize ROS, cells were incubated with MitoSOX (5 μM) for 30 min. Next, cells were resuspended in 500 μl of PBS containing 2% fetal bovine serum. The analysis was performed immediately in the BD FACS Verse. MFI (median fluorescence intensity) from non stained samples was used to normalize for each cell. MFI normalized from control (pLKO-empty) cells was set as 100%. The graph represents the relative fluorescence of the NEK10 depleted cells to control.

### Citrate synthase assay

Citrate synthase activity of HeLa pLKO, HeLa pLKO-sh89, HeLa pLKO-sh90 was measured and analyzed in a 96-well microplate according to Oroboros Instruments [30] with some modification. For each reaction, 6 μg of whole-cell extracts were incubated with 0.1 M Tris-HCl buffer (pH 7.1), 250 mM oxaloacetate, 100 mM DNTB, and 50 mM acetyl-coenzyme A. Absorbance at 412 nm was monitored for 5 min at 30 °C. The plate was read on a Eon™ Microplate Spectrophotometer - BioTek. Enzyme activity, in international units (IU, mU/mL), was calculated using the molar absorptivity coefficient of TNB (13.6 mM^− 1^ cm^− 1^) and an optical path length for the total volume of the reaction (0.527 for 0.2 mL). The data were normalized for protein concentration, and the results are presented in nmol/min/μg of protein.

### Long extension PCR

The total DNA of HeLa pLKO, HeLa pLKO-sh89, HeLa pLKO-sh90 was extracted using DNeasy Blood and Tissue kit (Qiagen), following the specifications of the manufacturer. The amplification of the short nuclear fragment, was performed following the reaction for Taq DNA Polymerase (Invitrogen, Cat# 10342–053). The nuclear gene (177 bp) amplified was HPRT1 (Hypoxanthine Phosphoribosyltransferase 1), using the oligos: Forward: 5′-TGACATGTGCCGCCTGCGAG-`3 and Reverse: GTGGTCGCTTTCCGTGCCGA. Long mitochondrial fragment amplification was achieved with the AccuPrime Taq kit (Invitrogen, Cat# 12346086). The mitochondrial fragment (16,540 bp) was amplified using the oligos: 5′-TGAGGCCAAATATCATTCTGAGGGGC-`3 and 5′- TTTCATCATGCGGAGATGTTGGATGG-`3. Intensity of band areas was measured using the ImageJ software. The measured intensity of the long fragment was divided by that of the short fragment.

### Statistical analysis

One-Way ANOVA with post-hoc Bonferroni, Tukey’s Multiple Comparison Test or with Student t-test unpaired two-tailed analysis was applied, to perform statistical analysis. The software used was GraphPad Prism version 7 (Graph Pad Software, San Diego, CA, USA). Graphs are shown as the mean ± S.E.M. and values of *p* < 0.05 were considered statistical significant and represented by *.

## Results

### NEK10 interactome

The interaction between proteins can be modulated by post-translation modifications, such as phosphorylation, and depends critically on the cellular compartmentalization [31]. Other members of the NEK family, including NEK4, NEK6 and NEK7 are considered Hub proteins (i.e. proteins with a large number of interactors) [5, 16, 32, 33]. To extend our knowledge about NEK10 interactors, we performed an interaction screen, based on immunoprecipitation followed by a mass spectrometry analysis (IP-LC-MS/MS). For that, HEK293T cells were transfected with p3XFLAG-CMV-7.1- (FLAG control) or p3XFLAG-CMV-7.1 (FLAG-NEK10). After 48 h of transfection, cells were treated or not (control) with Zeocin for 3 h. As a member of the Bleomycin family of antibiotics, Zeocin binds to and intercalates into DNA, leading to DNA double strand breaks (DSBs), predominantly via ROS formation [34]. Trypsin-digested peptides from NEK10-protein co-immunoprecipitated complexes and controls were identified through liquid chromatography mass spectrometry. Interactions were considered positive if protein identification occurred in two or more samples and if it occurred in one or less FLAG-control immunoprecipitates.

**Fig. 1:**
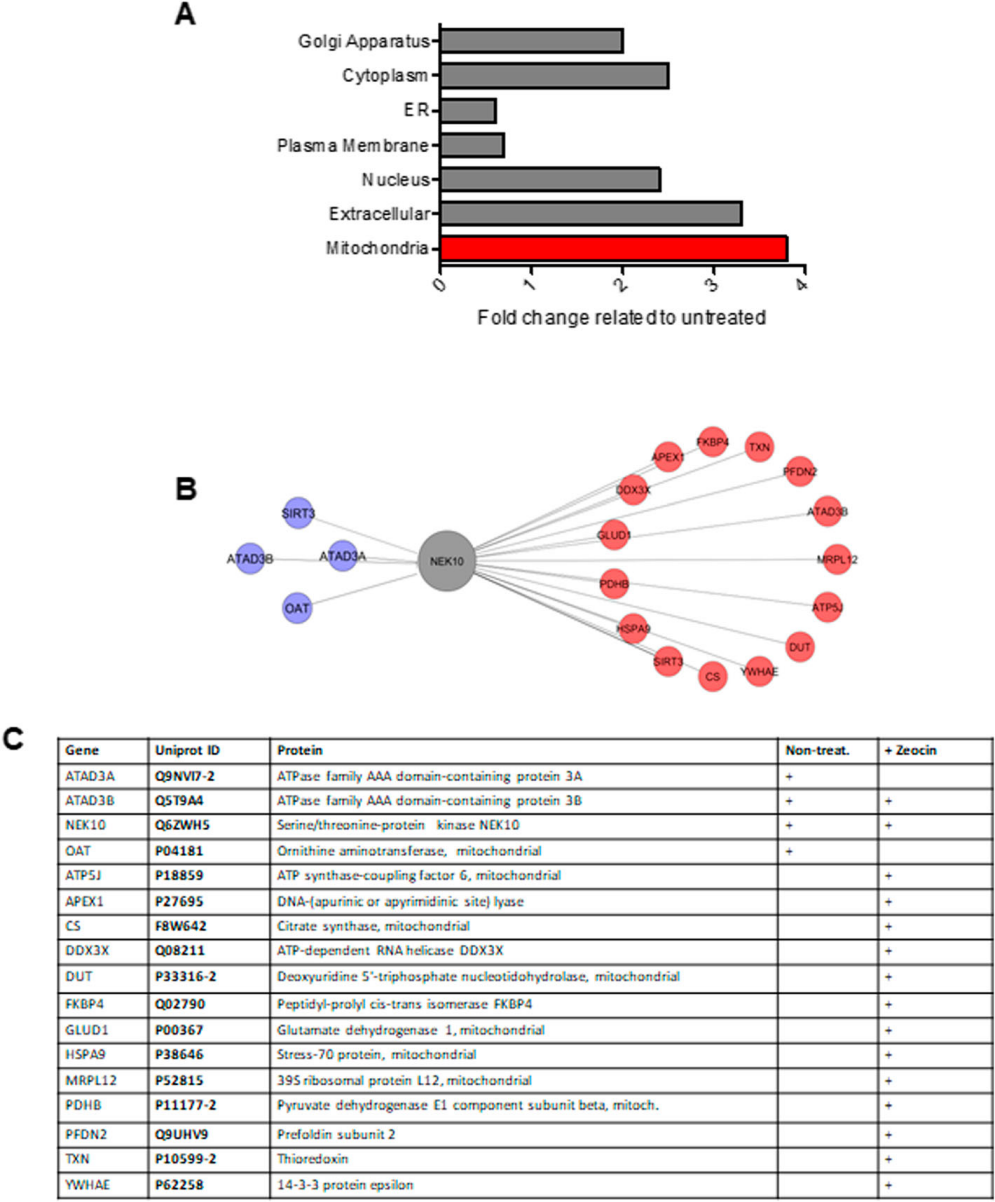
NEK10 interacts with mitochondrial protein partners. Interaction network of human NEK10 with partners identified by IP-LC-MS/MS reveals mitochondrial proteins enrichment after zeocin treatment. The proteomic data retrieved from IP-LC-MS/MS was submitted to the Integrated Interactome System (IIS) platform (National Laboratory of Biosciences, Campinas, Brazil) [22]. The protein-protein interaction network (PPI) was generated using Cytoscape software [24]. **a** The fold change in the number of the proteins found after zeocin treatment, relative to before treatment, for the main cellular compartments enriched in the network is shown in the graph. ER = endoplasmic reticulum, **b** Protein network showing mitochondrial proteins found in samples without treatment (purple nodes) and with zeocin treatment (red nodes). Clusters were made according to the Selected Cellular Component. SIRT3 is a protein interactor retrieved from the databank by IIS platform [22]. **c** Table showing mitochondrial proteins found in untreated and zeocin treated samples

IP-LC-MS/MS retrieved 59 interaction partners from different cellular compartments in untreated cells (Supporting Table 1). The localization of the detected proteins enriched for the cytoplasm (20), membrane (17), nucleus (9), endoplasmic reticulum (5), golgi apparatus (1) and extracellular (3) (Supporting Figure S1 A). Interestingly, after zeocin treatment, the number of interactors increased considerably to 118 proteins, among which partners from the mitochondrial compartment scaled up to three times (Supporting Figure S1 B). Specifically, mitochondrial proteins detected in the FLAG-NEK10-control were: SIRT3, ATAD3A, ATAD3B, OAT (Fig. 1a and c) and partners identified after genotoxic treatment included: FKBP4, TXN, PFDN2, ATAD3B, MRPL12, ATP5J, DUT, YWHAE, CS, SIRT3, HSPA9, PDHB, GLUD1, DDX3X, APEX1 (Fig. 1b – red cluster, and 1c). This result indicates that NEK10 is potentially involved in a mitochondria related response after zeocin-induced cellular stress.

**Fig. 2:**
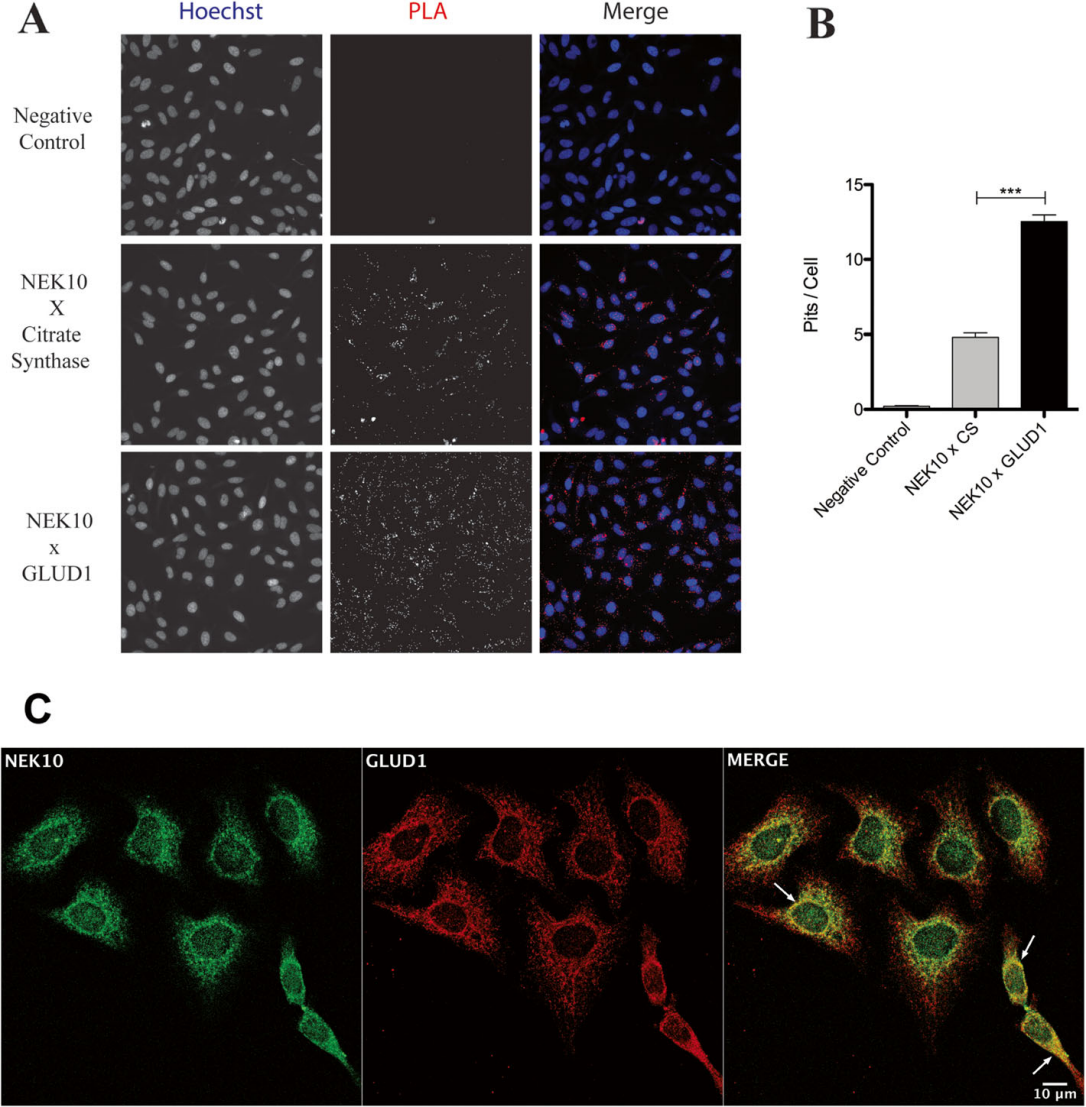
NEK10 interacts with GLUD1. **a** Proximity ligation assay of NEK10 vs. GLUD1/CS. HeLa pLKO cells were incubated with NEK10 (mouse or goat), GLUD1 (goat) and CS (mouse), and incubated with anti-mouse plus and anti-goat minus PLA reagents, followed by ligation, amplification and far-red staining reagents; nuclei were stained with Hoechst 33342 reagent. **b** Quantitative analysis of the average number of NEK10 and GLUD1 (or CS) interaction, indicated as PLA spots per cell, is represented in the bar graph. The images were collected with the ImageXpress, Micro Confocal High Content Image System (Molecular Devices) and the data were obtained with the MetaXpress software by using the transfluor module. One way ANOVA was used followed by the Bonferroni post hoc test. *** = *P* < 0.001. **c** Colocalization of NEK10 and Glutamate Dehydrogenase- 1 (GLUD1). Confocal images of HeLa cells stained with ant-NEK10 (in green) and anti-GLUD1 (in red) showing colocalization (merge, yellow) in the perinuclear region (arrows). Cells were visualized using a confocal Zeiss LSM 510, with a 63x oil objective, 1 air unit pinhole, 1024 × 1024 pixels. Scale bars: 10 μm. Images were analyzed using FIJI software [26]

We decided to further investigate Glutamate dehydrogenase 1 (*GLUD1*), a NEK10 interactor candidate, detected from the MS analysis. For that, we employed two different approaches: proximity ligation assay (Fig. 2a and b), and confocal immunofluorescence microscopy (Fig. 2c). The proximity ligation assay showed a interaction index that suggests a NEK10/GLUD1 complex (Fig. 2a) by the number of interactions (number of red spots) per cell.

Moreover, we tested citrate synthase (CS) protein, also detected as a NEK10 candidate partner in MS analyses. Comparing both data, we observed a higher quantity of spots for NEK10/GLUD1 compared to NEK10/CS. The negative control samples were performed with the rolling-circle amplification reaction but without primary antibodies or without the probes.

The confocal immunofluorescence microscopy also shows the co-localization between NEK10 and GLUD1 with a Pearson’s correlation coefficient of 0.8 (±0,008) (Fig. 2c). Both approaches suggest that NEK10 and GLUD1 could be interaction partners. Future experiment will address further details of these interactions.

### NEK10 localizes in mitochondria

**Fig. 3:**
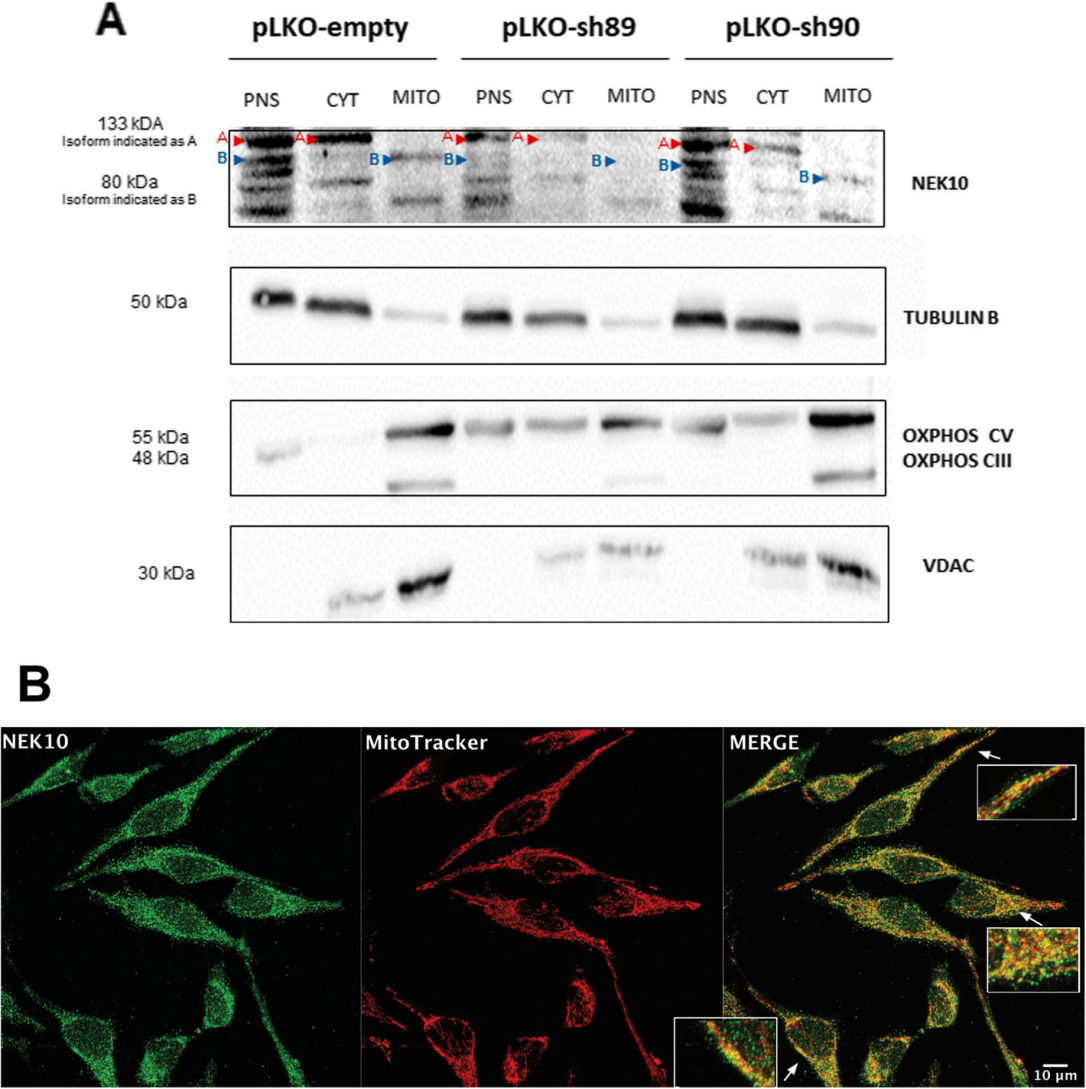
NEK10 is localized in the mitochondria. **a** Mitochondrial fractionation assay. Mitochondria from HeLa pLKO, HeLa pLKO-sh89 and HeLa pLKO-sh90 cells were isolated and NEK10 was analyzed by Western blot using anti-NEK10 antibody. Postnuclear supernatant (PNS), cytosol (CYT) and mitochondrial (MITO) fractions were analyzed with anti-Tubulin B, anti-OXPHOS and anti-VDAC antibodies, for fraction purity. The letter A and the red arrows indicate the 133 kDa isoform, present in PNS and CYTO. The letter B and the blue arrows indicate the 80 kDa isoform, present in the mitochondrial fraction (MITO). **b** Confocal images of NEK10 in mitochondria. HeLa pLKO-empty cells were stained using anti-NEK10 antibody (green) and the mitochondria were visualized with MitoTracker DeepRed for 20 min (red). Cells were analyzed in a confocal Zeiss LMS 510 microscope, using 63x oil objective, 1 air unit pinhole, 2048 × 2048 pixels. Scale bars: 10 μm. Images were analyzed using FIJI software [26]. Insets are enlargements of the outlined areas

Based on the finding that NEK10 interacts with multiple proteins with mitochondrial localization we next investigated, whether NEK10 localizes to the mitochondria. First, we performed a mitochondrial fractionation assay in HeLa pLKO, HeLa pLKO-sh89 and HeLa pLKO-sh90 cells (Fig. 3a). The data showed that NEK10 is present in both the cytosol (CYT) and mitochondrial (MITO) fractions for all three cell lines. The CYT and MITO fractions show detection of NEK10 bands of several molecular weights, suggesting different isoforms. For better visualization, the 133 kDa isoform of NEK10 is assigned with the letter A and red arrows (present in the PNS and CYTO extracts) while the 80 kDa isoform is represented with the letter B and blue arrows (MITO fraction). For NEK10-depleted cells (HeLa pLKO-sh89 and HeLa pLKO-sh90), comparing with HeLa pLKO, the MITO fractions show a reduction of the NEK10 expression (Fig. 3a). The corresponding band has a molecular weight of approximately 80 kDa. Marker proteins for cytoplasm (Tubulin B) and mitochondria fraction (OXPHOS, VDAC) were used to control the fractions.

We performed the same assay also for MRC5 and HEK293T cells (Supporting Figure S2 A and B, respectively), and again NEK10 was detected in the mitochondrial fractions. In these analyses, we used specific antibodies to confirm the purity of Postnuclear supernatant (PNS), cytosol (CYT) and mitochondrial (MITO) fractions. Interestingly, analyses by Western blotting (Supporting Figure S3) of the efficiency of depletion by shRNA, resulted in a clear decrease of the 80 kDa NEK10 band. This indicates that the mitochondrial NEK10 is around 40–60% depleted, whereas the cytoplasmic NEK10 is about 60–80% depleted (Fig. S2A-C). We believe that the bands visualized in the Western blot, correspond to NEK10 isoforms as described on the NCBI and UniProt websites: UniProtKB - Q6ZWH5 (NEK10_HUMAN) [35, 36].

The specificity of mouse anti-NEK10 antibody was tested by using immunofluorescence assays (Supporting Figure S4). HeLa cells transfected with FLAG-NEK10 presented an increase in fluorescence with mouse NEK10 antibody staining. The plasmid pcDNA6-FLAG-Ki-1/57 [19] was used as a negative control. The goat anti-NEK10 antibody was used as previously described [9]. The rabbit anti-NEK10 datasheet further shows the specificity for the 80 kDa NEK10 isoform.

Confocal immunofluorescence microscopy was employed as a second approach to confirm whether NEK10 is located to the mitochondria. HeLa pLKO-empty cells were stained with anti-NEK10 antibody (in green) and the mitochondria with MitoTracker DeepRed (in red) (Fig. 3b). The co-localization of NEK10 with the mitochondrial marker (with a Pearson’s correlation coefficient of 0,54 ± 0,01) provides additional support for NEK10’s localization to the mitochondria.

### The essential role of NEK10 for mitochondrial morphology

**Fig. 4:**
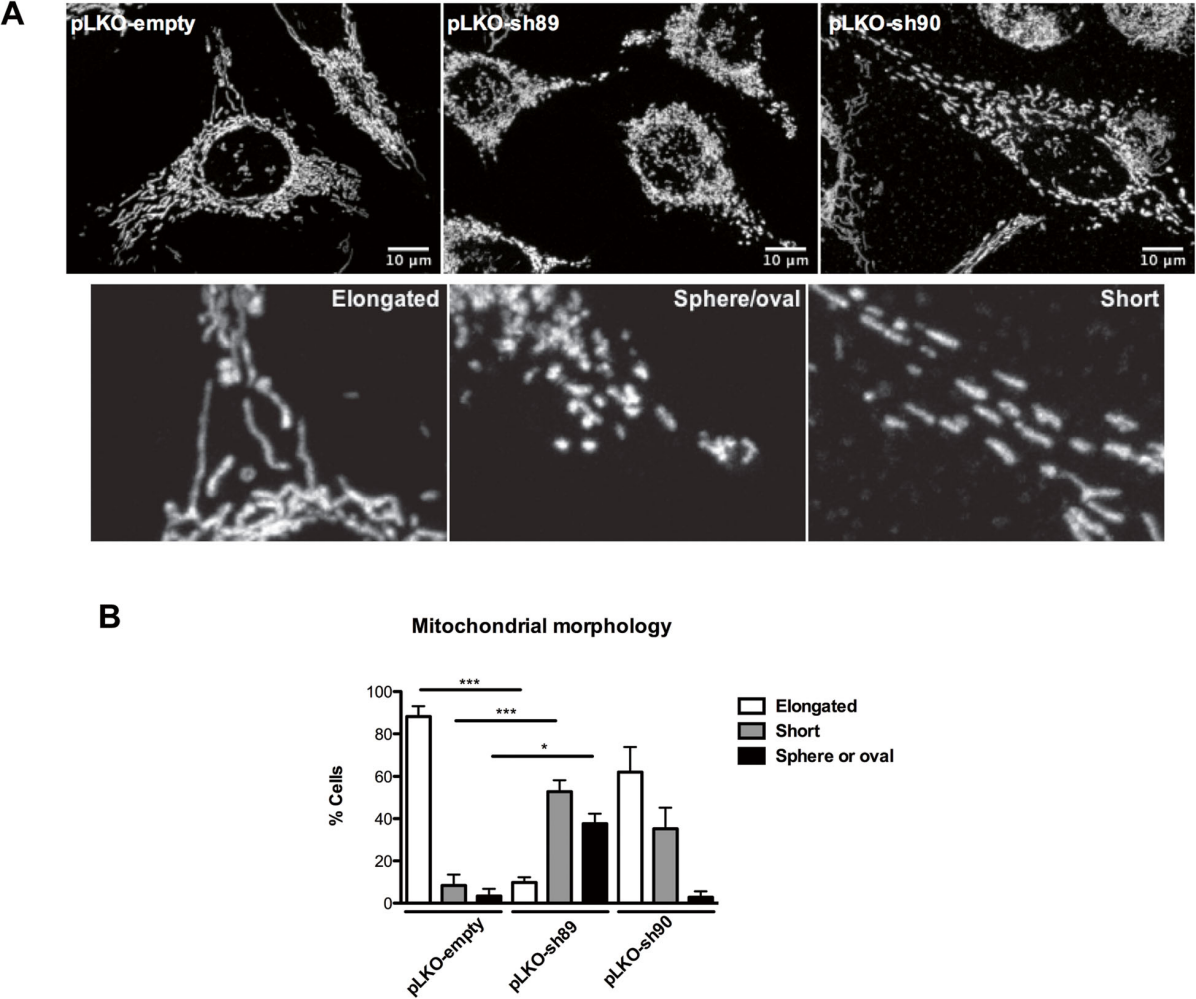
The essential role of NEK10 for mitochondria morphology. **a** Representative confocal images of mitochondrial morphology in HeLa cells shRNA for Nek10 (pLKO-sh89 and pLKO-sh90) or control (pLKO-empty) stained with MitoTracker DeepRed for 20 min. Micrographs were acquired from live cells, using 63× oil objective, 1 air unit pinhole, 1024 × 1024 pixels. Scale bars: 10 μm. **b** Quantitative analysis of mitochondrial morphology is shown. Cells were classified on the basis of their mitochondrial morphology: oval/spheres, short (< 6 μm) or long (> 6 μm) mitochondrial filaments. At least 30 cells/sample were counted. One way ANOVA was used followed by the Bonferroni post hoc test. * = *p* < 0.05; *** = *P* < 0.001

To examine the role of NEK10 in mitochondria, we performed confocal immunofluorescence of HeLa pLKO, HeLa pLKO-sh89 and HeLa pLKO-sh90 cells. All cell lines were stained with MitoTracker DeepRed for 20 min (Fig. 4a). The graph (Fig. 4b) shows the quantification of mitochondrial length detected in confocal microscopy: Fragmented oval/spheres, fragmented short (< 6 μm) mitochondrial filaments or Tubular [elongated (> 6 μm) mitochondrial filaments]. NEK10-depleted cells (specially HeLa pLKO-sh89) showed more fragmented than tubular mitochondrial filaments comparing to the control cells (HeLa-pLKO).

**Fig. 5:**
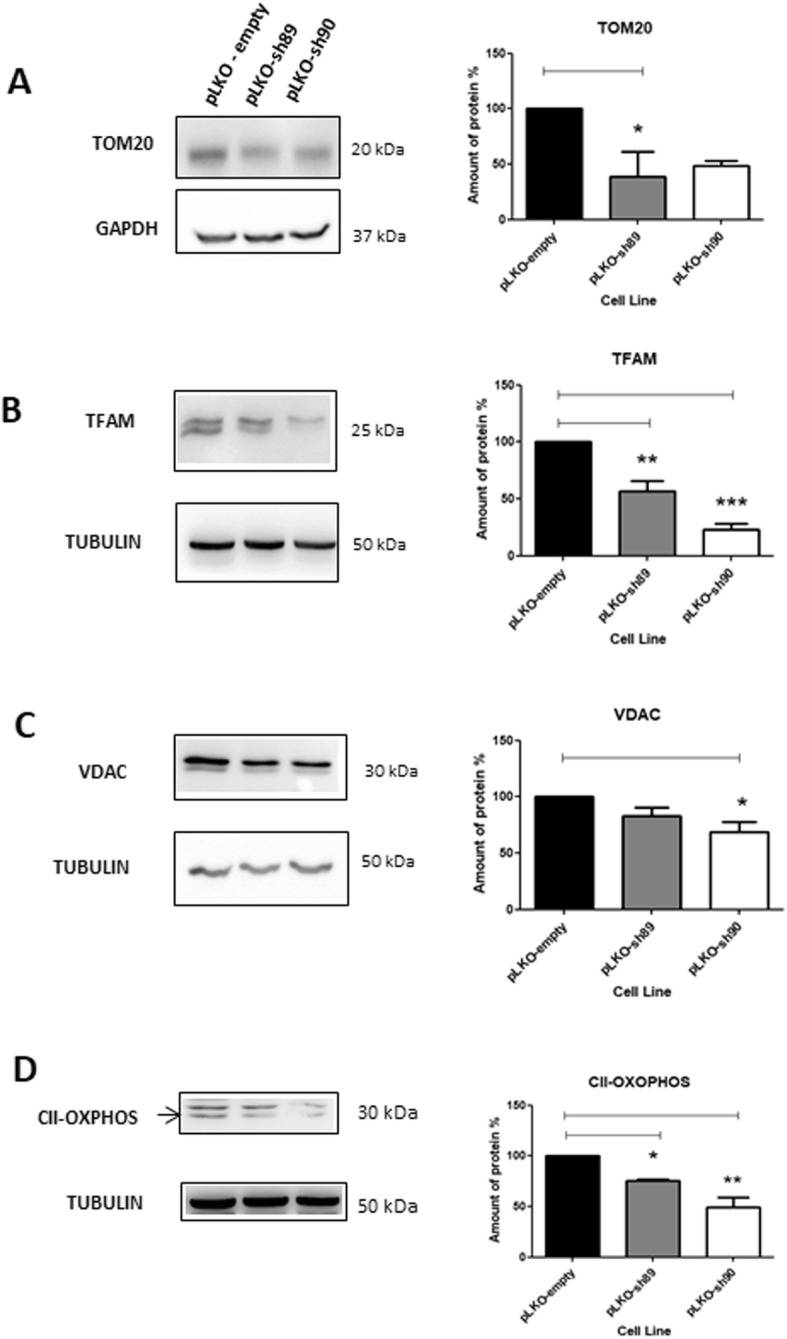
NEK10 depleted cells show a decrease in mitochondrial mass. Expression of four different mitochondrial marker proteins (**a** TOM20, **b** TFAM, **c** VDAC and **d** OXPHOS) was analyzed from HeLa pLKO, HeLa pLKO-sh89 and HeLa pLKO-sh90 total lysates. For immunoblotting, the following antibodies were used: anti-TOMM20, anti-mtTFAM, anti-VDAC1 and anti-OXPHOS. The quantification graphs on the right show averages from 4 independent experiments. The statistical analyses were performed using One-Way ANOVA followed by Tukey’s Multiple Comparison Test. (* = *P* < 0.05; ** = *P* < 0.01; *** = P < 0.001)

To complement the mitochondrial morphology study, we quantified some mitochondrial proteins in HeLa pLKO, HeLa pLKO-sh89 and HeLa pLKO-sh90 cells total lysates (Fig. 5a-d). Four proteins were investigated: TOM20, TFAM, VDAC and OXPHOS Complex II. All of these proteins showed decreased bands intensity in depleted NEK10 cells (HeLa pLKO-sh89 and HeLa pLKO-sh90), indicating a reduction in mitochondrial mass that may or may not be related to the mitochondrial morphology features of Fig. 4.

### NEK10 knock down compromises mitochondrial respiration

Next, we investigated the effects of NEK10 knock down on oxygen consumption/respiration, as this is one of the mitochondria’s main function. For that, we employed OROBOROS Oxygraph-2 k to analyze the respiration rates of HeLa pLKO, HeLa pLKO-sh89 and HeLa pLKO-sh90 cell lines in the presence of inhibitors of the mitochondrial respiratory chain.

**Fig. 6:**
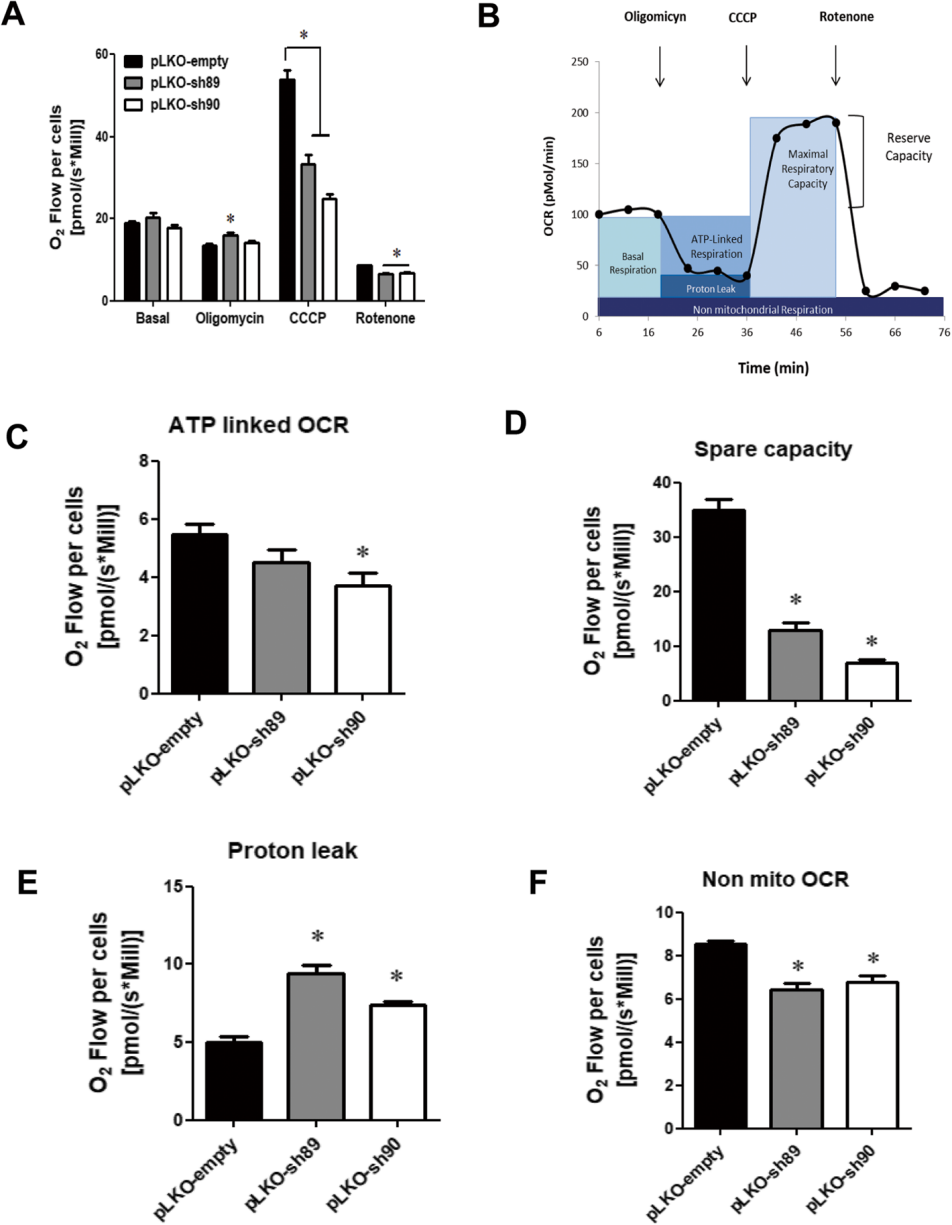
The mitochondrial respiratory chain is negatively affected by the depletion of NEK10. **a** The graphic shows O_2_ Flow per cells by basal respiration and after treatments (Olygomycin, CCCP and Rotenone) for HeLa pLKO-empty, HeLa pLKO-sh89 and HeLa pLKO-sh90. **b** Visualization of experimental oxygen consumption rate (OCR) data type nomenclature, adapted from Rose and coworkers [56]. **c** Measurement of ATP-Linked to Oxygen Consumption Rate (OCR). **d** Measurement of spare capacity. **e** Measurement of proton leak. **f** Measurement of non-mitochondrial oxygen consumption (non mito OCR). The analyses were performed in quadruplicate. The statistical analyses were performed using One-Way ANOVA followed by Tukey’s Multiple Comparison Test. (* = P < 0.05)

Figure 6a and Supporting Figure S5 show the typical measurements of oxygen consumption. The basal mitochondrial respiration was similar between control and NEK10-depleted cells. However, after CCCP addition (a chemical mitochondrial protonophore) the maximal respiration capacity of pLKO was almost two times higher than that of the NEK10-depleted cells. This finding, therefore, is clearly indicating a lower mitochondrial electron transfer capacity in NEK10-depleted cells. The levels of oxygen consumption for NEK10-depleted cells were also significantly reduced in response to rotenone, a mitochondrial inhibitor of complex I.

To evaluate in more details the effects of NEK10 depletion, the ATP-linked oxygen consumption rate (OCR), spare capacity and proton leak (as illustrated in Fig. 6b) were analyzed. OCR measured by the difference in basal respiration rate after oligomycin addition indicate that ATP-linked OCR is reduced in NEK10 depleted cells compared to the control (Fig. 6c).

In Fig. 6d, we observed that control cells have a higher spare capacity compared with NEK10-depleted cells. These results indicate that NEK10 depleted cells are prone to exhaustion of the respiratory reserve capacity.

Proton leak effectively is measured by the respiratory chain activity in the presence of oligomycin [37]. The mitochondrion has a basal proton leak but agents that provoke the permeability of the mitochondrial inner membrane can also induce it. The basal proton leak may have a role in cell protection [38]. NEK10-depleted cells have a more pronounced proton leak (Fig. 6e) than control cells. When the non-mitochondrial oxygen consumption (non mito OCR) was analyzed, control cells clearly consumed more oxygen than depleted cells (Fig. 6f).

In summary, these results indicate that NEK10 can regulate OXPHOS, as NEK10 depletion affects ATP-linked OCR, spare capacity, proton leak and non-mitochondrial OCR. The data further suggest that NEK10 depletion may alter negatively the mitochondria respiration, which may occur directly or indirectly through its interactors, either via protein interaction or phosphorylation mechanisms.

### NEK10 knock down changes mitochondrial reactive oxygen species (ROS) levels and decreases citrate synthase activity

**Fig. 7:**
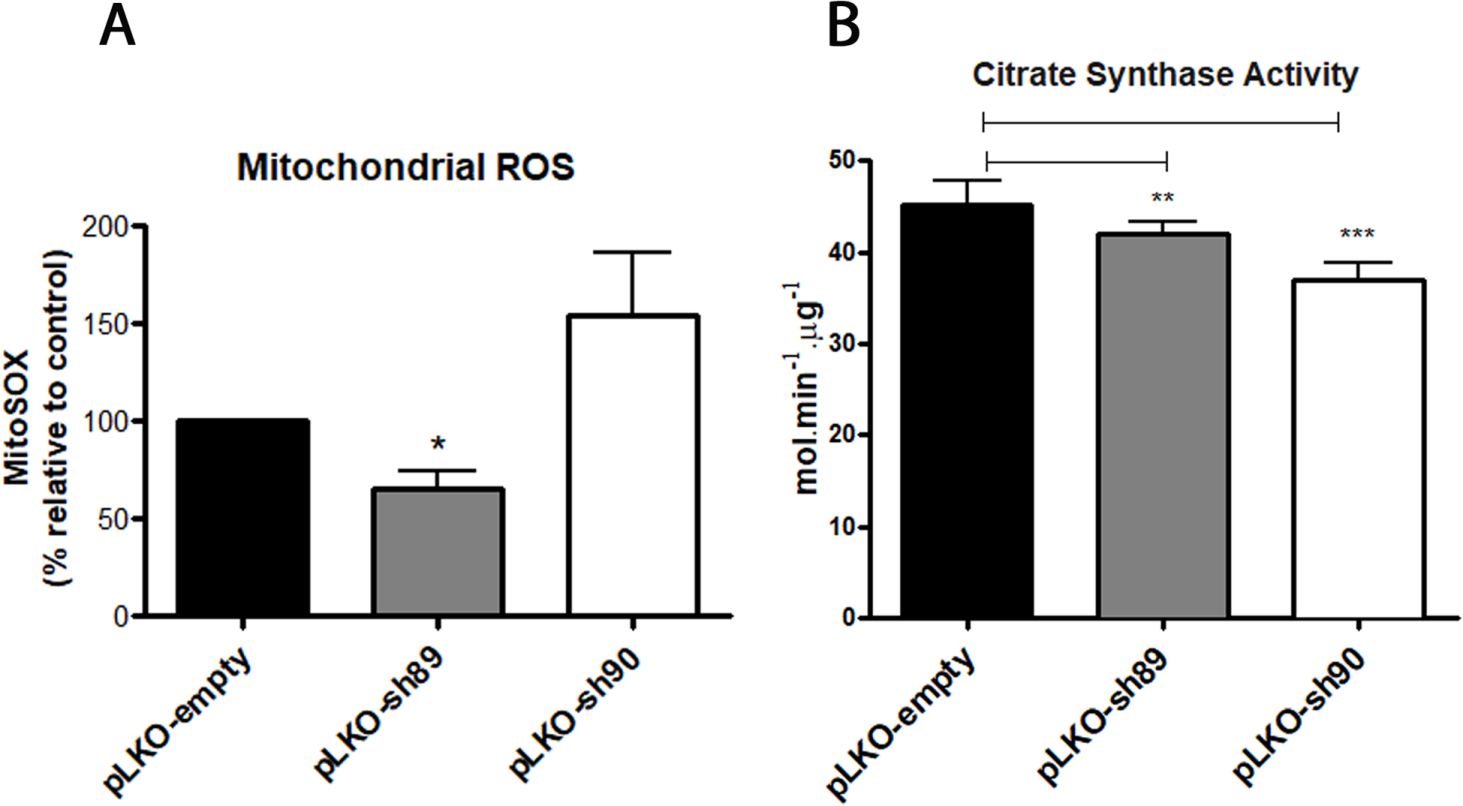
The knock down of NEK10 changes mitochondrial reactive oxygen species (ROS) levels, and decreases citrate synthase activity. **a** Mitochondrial superoxide from HeLa pLKO, HeLa pLKO-sh89 and HeLa pLKO-sh90 cells was measured, using MitoSOX probe, through flow cytometry. Results represent the percentage of median fluorescence from at least three independent experiments relative to control (pLKO-empty). The statistical analyses were performed using GraphPad Prism software using Student t-test unpaired two-tailed analysis: (*) *p* < 0.05. **b** Total protein extract of HeLa pLKO, HeLa pLKO-sh89 and HeLa pLKO-sh90 cells was used to measure the citrate synthase activity with acetyl-CoA. The colorimetric assay was performed using a spectrophotometer. The graph shows analyses from *n* = 5 independent experiments. One-Way ANOVA followed by Tukey’s Multiple Comparison Test was used for statistical analyses (* = P < 0.05; ** = P < 0.01; *** = P < 0.001)

Since NEK10 knock down affected mitochondrial morphology and respiration metabolism we performed further assays related to mitochondrial functions. Using flow cytometry, mitochondrial ROS from HeLa pLKO, HeLa pLKO-sh89 and HeLa pLKO-sh90 cells was measured using MitoSOX (Fig. 7a). The data show that HeLa pLKO-sh89 has lower ROS levels compared with the control cells. That could be related to the high mitochondria fragmentation found in these cells maybe due to possible mitophagy. However, HeLa pLKO-sh90 cells presented increased ROS levels (Fig. 7a) and a less pronounced mitochondria fragmentation.

As observed in Fig. 7b, the Citrate Synthase activity was measured for HeLa pLKO, HeLa pLKO-sh89 and HeLa pLKO-sh90 cell lines. Mitochondrial Citrate Synthase plays a central role in aerobic energy production; also, it is the first enzyme of the Tricarboxylic Acid Cycle (TCA) [39]. The data show a significant decrease of Citrate Synthase activity in the NEK10 depleted cells (HeLA pLKO-sh89 and HeLA pLKO-sh90). This suggests a role of NEK10 in aerobic energy production that may impact mitochondrial mass in these cells, since mitochondrial mass is regulated by the energy production levels in the cell.

### NEK10 silencing increases mtDNA damage and mtDNA copy number

**Fig. 8:**
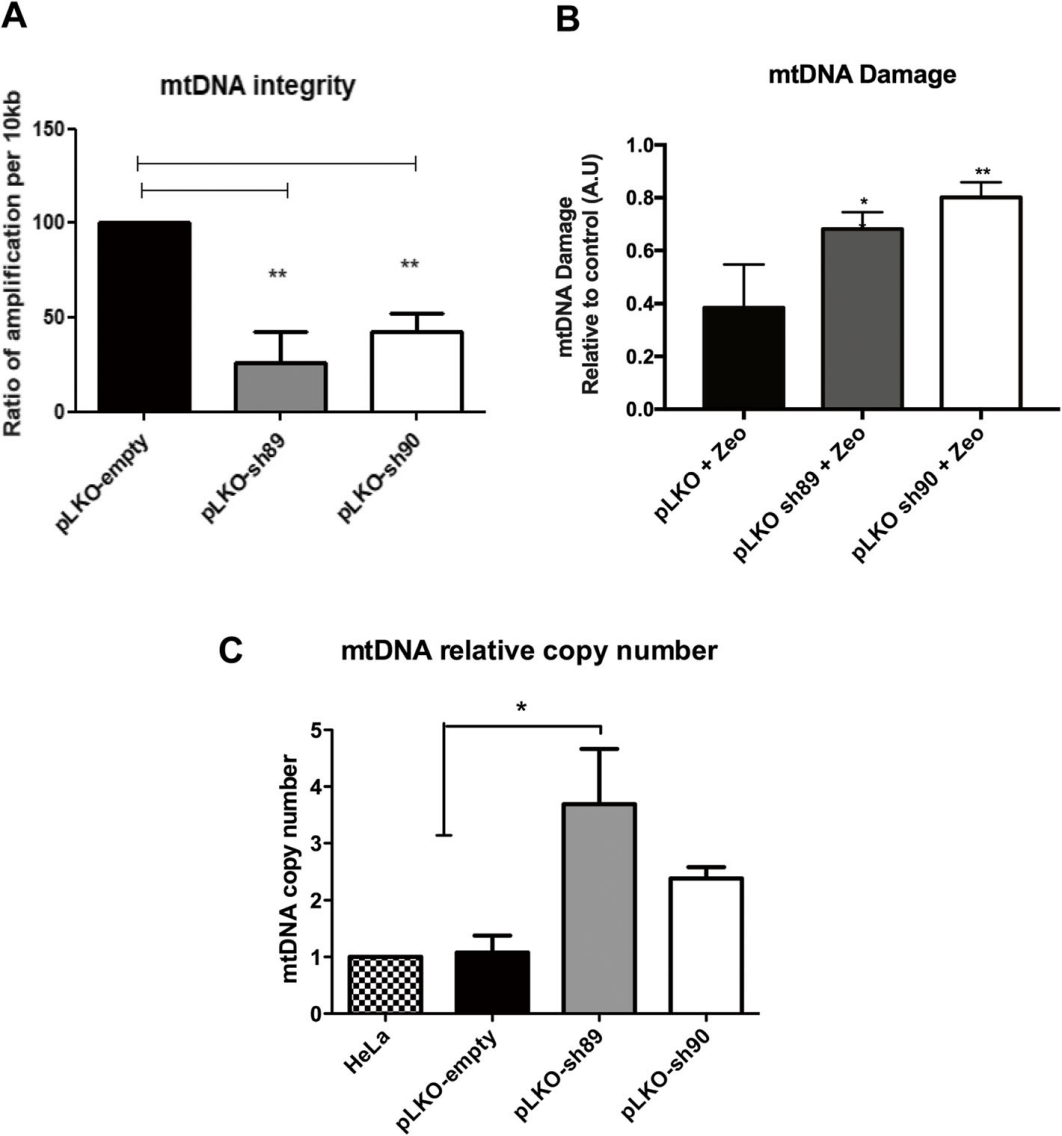
Mitochondrial DNA (mtDNA) analysis of HeLa pLKO-empty, HeLa pLKO-sh89 and HeLa pLKO-sh90 cells. **a** The mtDNA integrity of NEK10 depleted and control cells was analyzed by Long-Extension PCR and the ratio of the amplification is shown in the graph in basal conditions. **b** The mtDNA integrity of cells treated with 300 μg/ml zeocin treatment for 3 h was evaluated by long-extension PCR. Results show the difference in mtDNA damage of cells with zeocin treatment from the mtDNA damage under basal conditions. The statistical analysis is a comparison to the control wild type cells. **c** The number of mitochondrial DNA copy number by the different cells analyzed by Real-Time quantitative polymerase chain reaction (qPCR). Both graphs show analyses from *n* = 3 independent experiments. Statistical analyses were made by One-Way ANOVA and the Tukey’s Multiple Comparison Test. * = P < 0.05; ** = P < 0.01

The effect of NEK10 depletion on mitochondrial DNA integrity (mtDNA) and relative mtDNA copy number was also investigated. For mtDNA damage assays, we employed Long-extension PCR. The NEK10 depletion in HeLa pLKO-sh89 and HeLa pLKO-sh90 cells, decreased the ratio of amplification of mtDNA, which suggests that their mtDNA has more damage than that of the control cells (HeLa pLKO) (Fig. 8a). Also, we tested the mtDNA integrity in the presence of 300 μg/ml zeocin treatment for 3 h (Fig. 8b). We observed that compared to wild type cells the silencing of NEK10 results in a higher mtDNA damage, in the presence of zeocin. Finally, we also analyzed the mtDNA copy number, using Real-Time PCR with ND1 and HPRT primers, as the respective markers (Fig. 8c). Interestingly, NEK10 depletion increased 1.7 fold the levels of mtDNA, suggesting that NEK10 is involved in the controling mtDNA content, either by controlling DNA replication or mitochondria numbers. These results suggest, that the lack of NEK10 may result in mitochondrial homeostasis impairment, resulting in mtDNA damage and unbalance mtDNA levels.

## Discussion

Among the NEK10 protein partners localized in mitochondria, we identified Citrate Synthase (CS), glutamate dehydrogenase (GLUD1), pyruvate dehydrogenase and others (Figs. 1 and 2 and Supporting Tables S1). That could be an insight that indicates that NEK10 has roles in mitochondria.

Interestingly, an increase in the number of mitochondrial protein partners for NEK10 was observed after zeocin treatment (Fig. 1). Data from the literature showed that zeocin treatment in HeLa cells induces apoptosis in a caspase pathway dependent way [40]. To confirm one of those interactors, we performed proximity ligation assay (Fig. 2a and b). Furthermore, employing confocal immunofluorescence analyses, we detected the co-localization of NEK10 and GLUD1 (Fig. 2c). GLUD1 is a mitochondrial matrix enzyme that catalyzes the conversion of glutamate to α-ketoglutarate and ammonia, while reducing NAD(P)^+^ to NAD(P)H. This contributes to both Krebs cycle anaplerosis and energy production. Furthermore, GLUD1 has a role in the redox homeostasis [41].

When we performed cell fractionation, we found NEK10 in the mitochondria fraction of HeLa (Fig. 3a), MRC5 (Supporting Figure S2A), and HEK293T cells (Supporting Figure S2B). Interestingly, the NEK10 levels in the mitochondria fractions from depleted HeLa shNEK10–89 and shNEK10–90 were decreased. Also, the NEK10 mitochondrial localization was confirmed employing confocal immunofluorescence analyses from HeLa pLKO-empty cells (Fig. 3b); thereby supporting the idea that NEK10 is present in the mitochondria.

Our data in Fig. 4 show that NEK10-depleted cells (HeLa pLKO-sh89) have more fragmented than tubular mitochondrial filaments, compared to the control cells (HeLa-pLKO). The mitochondrial fragmentation is related to pre-mitosis events, a nutrient-rich environment (associated with an increase in membrane potential and proton leak), or can be associated with mitochondrial impairment, elevated stress levels and cell death. On the other hand, tubular mitochondrial filaments confer protection against phagophore engulfment when autophagy is triggered [42].

Mitochondrial proteins in HeLa pLKO, HeLa pLKO-sh89 and HeLa pLKO-sh90 cells from total lysates were quantified (Fig. 5). Mitochondrial outer membrane translocase complex, TOM20 is part of the TOM complex and is a major import receptor that recognizes the mitochondrial sequence [42]. TFAM is involved in many functions: mtDNA transcription, mtDNA maintenance and replication and mtDNA repair [43, 44]. The voltage-dependent anion channel (VDAC) is present in the outer mitochondrial membrane (OMM) and it participates in mitochondrial permeabilization [45]. Mitochondrial complex II (CII) is also known as succinate:ubiquinone oxidoreductase (SQR) or succinate dehydrogenase (SDH). The four subunits of CII are encoded by the nuclear genome and have roles in energy production [46]. To evaluate the role of NEK10 in cellular bioenergetics, we analyzed mitochondrial oxygen consumption in intact cells. Our results show that the depletion of NEK10 compromised mitochondrial respiration (Fig. 6).

In Fig. 6d, we observed that control cells have a larger spare capacity compared with NEK10-depleted cells. A decrease in spare capacity has been related to pathologies that affect tissues that require a large amount of energy, such as the brain, heart, and skeletal muscle [47]. These results indicate that NEK10 depleted cells are prone to exhaustion of the respiratory reserve capacity.

Upon depletion of NEK10, cells presented reduced non-mitochondrial oxygen consumption compared to control cells. Although mitochondria are responsible for the major part of cellular respiration, cells like neutrophils can activate non-mitochondrial oxygenases. Also in macrophages, the activity of non-mitochondrial NADPH oxidases may be the major source for cellular oxygen consumption. It is possible to measure non-mitochondrial oxygen consumption using a mitochondrial electron transport chain inhibitor such as rotenone. In other cells, desaturation and detoxification enzymes are responsible for 10% of the non-mitochondrial oxygen consumption [48].

Our flow cytometry data (Fig. 7a) showed a possible relationship between mitochondrial fragmentation and mitochondrial ROS production. The decrease of ROS levels in NEK10-depleted cells (HeLa sh89) compared to the control cells (HeLa pLKO empty), may be related to the high degree of mitochondrial fragmentation observed in the HeLa sh89 cells (Fig. 4a and b).

Furthermore, we observed a decrease of CS activity in NEK10-depleted cells (HeLa sh89 and sh90) compared to control cells (HeLa pLKO) (Fig. 7b). It is known, that the lack of CS activity can lead to respiratory defects [39]. Lin and coworkers reported, that cells with CS knock down exhibited defects in respiratory activity and a considerable decreases in ATP production [49]. Our results also showed a decrease in the respiratory activity (Fig. 6) in parallel with the decrease in CS activity. Interestingly, CS was identified as a possible NEK10 partner in our MS proteomics analysis and the decrease in CS activity in NEK10 depleted cells might indicate a regulatory connection between these two proteins, either directly or indirectly.

The mammalian cell contains several hundreds to thousands of mitochondria and each mitochondrion has 2–10 copies of mtDNA, a circular, double stranded DNA that encodes 37 genes essential for mitochondrial functions. This variation of mitochondrial numbers is tissue and age dependent [50]. Also, there is evidence that suggests that mtDNA copy number increases after exposure to DNA damaging agents [51]. We identified an increase in mtDNA copy number (Fig. 8c), along with a reduction of mtDNA integrity in NEK10-depleted cells (HeLa sh89 and HeLa sh90), in both the abscence and presence of zeocin (Fig. 8a, b).

We observed a 1.7-fold increase in mtDNA index after silencing of NEK10 (Fig. 8b, c). Studies suggest that alterations in mtDNA copy number (increase or decrease) are potentially involved with tumorigenesis [52–55]. There is an increase of mtDNA copy number in patients with glioma [52], patients with an elevated risk of breast cancer [53], and in endometrial adenocarcinoma cells [54]. Mitochondrial genome mutations are correlated with mitochondrial disorders (mitochondrial cytopathies), affecting both nervous and muscular tissue [55].

## Conclusion

In summary, we reported here the NEK10 protein interactome and demonstrated its new roles in mitochondrial homeostasis. Silencing NEK10 expression changed mitochondrial morphology, increased the mtDNA damage and mtDNA content, potentiated cell death, led to increased ROS levels, and inhibited the mitochondrial respiration and citrate synthase activity. Our research opens new avenues for the study of NEK10 functions in the context of the underlying mechanisms in mitochondrial homeostasis such as respiration and related pathologies, including tumorigenesis.

## Supplementary information

**Additional file 1: Figure S1.** NEK10 interacts with mitochondrial partners. Interaction network of human NEK10 with proteins partners, identified by IP-LC-MS/MS. Tryptic-digested peptides from FLAG or FLAG-NEK10 immunoprecipitates were analyzed by mass spectrometry and protein partners were identified. The samples were untreated (A) or treated with zeocin (B). The proteomic data retrieved from IP-LC-MS/MS was submitted to the Integrated Interactome System (IIS) platform (National Laboratory of Biosciences, Campinas, Brazil) [Carazzolle et al., 2014] [22]. The protein-protein interaction network (PPI) was generated using Cytoscape software [Shannon et al., 2003] [24]. **Figure S2.** Mitochondrial fractionation. Mitochondria from A- MRC5 cells and B- HEK293T cells were isolated and the localization of NEK10 was analyzed by Western blot using anti-NEK10 antibody. Postnuclear supernatant (PNS), cytosol (CYT) and mitochondrial (MITO) fractions were analysed with anti-Lamin A/C, anti- GAPDH, anti-Tubulin A, anti-OXPHOS and anti-VDAC, to access fractionation purity. The letter A and the red arrows indicate the 133 kDa isoform present in PNS and CYTO. The letter B and the blue arrows indicate the 80 kDa isoform present in the mitochondrial fraction (MITO). **Figure S3.** Validation of NEK10 depletion in HeLa cells by shRNA. Two different pLKO-shRNAs were designed to target NEK10 (shNEK10–89 and shNEK10–90, named here as sh89 and sh90, respectively). A- Immunoblotting of HeLa pLKO, HeLa pLKO-sh89 and HeLa pLKO-sh90 cells lysates with anti-NEK10 antibody and anti-GAPDH antibody. B and C- The graphs B and C show the percentage of 133 kDa and 80 kDa NEK10 depletion, respectively. The quantification is shown from *n* = 5 independent experiments. The letter A and red arrows indicate 133 kDa NEK10 isoform. The letter B and blue arrows indicate 80 kDa NEK10 isoform. **Figure S4.** NEK10 antibody presented specificity. HeLa cells transfected with FLAG-Nek10 presented increased fluorescence intensity in the mouse anti-NEK10 antibody staining, demonstrating the antibody specify. pcDNA6-FLAG-Ki-1/57 was used as a negative control of increase in fluorescence intensity after staining with NEK10 antibody. **Figure S5.** Trace of oxygen consumption rate (OCR) for control and NEK10 depleted cells. OROBOROS Oxygraph-2 k was used to evaluate the respiration rate of HeLa pLKO-empty, HeLa pLKO-sh89 and HeLa pLKO-sh90 cells in the presence of substrates as well as inhibitor of the mitochondrial respiratory chain (rotenone) or ATP synthase (oligomycin). The mitochondrial uncoupling was induced by CCCP.

**Additional file 2: Table S1.** NEK10 protein interactors identified in HEK293T cells treated or not with zeocin by IP-MS/MS. Proteins marked with an asterisk: *: Retrieved from the databank by IIS platform [Carazzolle et al., 2014]. The mass spectrometry data have been deposited to the ProteomeXchange Consortium via the PRIDE partner repository with the dataset identifier PXD018294.

## Data Availability

All data generated or analyzed during this study are included in this published article, and its supplementary information files. The mass spectrometry data have been deposited to the ProteomeXchange Consortium via the PRIDE partner repository with the dataset identifier PXD018294.

## References

[1] Manning G, Whyte DB, Martinez R, Hunter T, Sudarsanam S (2002). The protein kinase complement of the human genom. Science.

[2] Davies JR, Osmani AH, De Souza CPC, Bachewich C, Osmani SA (2004). Potential link between the NIMA mitotic kinase and nuclear membrane fission during mitotic exit in aspergillus nidulans. Eukaryot Cell.

[3] Govindaraghavan M, Lad AA, Osmani SA (2014). The NIMA kinase is required to execute stage-specific mitotic functions after initiation of mitosis. Eukaryot Cell.

[4] De Souza CP, Osmani AH, Wu LP, Spotts JL, Osmani SA (2000). Mitotic histone H3 phosphorylation by the NIMA kinase in aspergillus nidulans. Cell.

[5] Meirelles GV, Perez AM, de Souza EE, Basei FL, Papa PF, Melo Hanchuk TD (2014). “Stop ne(c) king around”: how interactomics contributes to functionally characterize Nek family kinases. World J Biol Chem.

[6] Davies H, Hunter C, Smith R, Stephens P, Greenman C, Bignell G, et al. Somatic mutations of the protein kinase gene family in human lung cancer. 2005;65:7591–6. 10.1158/0008-5472.CAN-05-1855 16140923

[7] Antoniou AC, Beesley J, Mcguffog L, Sinilnikova OM, Healey S, Neuhausen SL, et al. Common breast cancer susceptibility alleles and the risk of breast cancer for BRCA1 and BRCA2 mutation carriers : implications for risk prediction. Cancer Res. 2010;70:9742–54. 10.1158/0008-5472.CAN-10-1907 PMC2999830 21118973

[8] Moniz LS, Stambolic V (2011). Nek10 mediates G2/M cell cycle arrest and MEK autoactivation in response to UV irradiation. Mol Cell Biol.

[9] Porpora M, Sauchella S, Rinaldi L, Delle Donne R, Sepe M, Torres-Quesada O, et al. Counterregulation of cAMP-directed kinase activities controls ciliogenesis. Nat Commun. 2018;9:1224. 10.1038/s41467-018-03643-9 PMC5964327 29581457

[10] Friedman JR, Nunnari J (2014). Mitochondrial form and function. Nature..

[11] Vakifahmetoglu-Norberg H, Ouchida AT, Norberg E. The role of mitochondria in metabolism and cell death. Biochem Biophys Res Commun. 2017;482:426–31. 10.1016/j.bbrc.2016.11.088 28212726

[12] Chen Y, Craigen WJ, Riley DJ (2009). Nek1 regulates cell death and mitochondrial membrane permeability through phosphorylation of VDAC1. Cell Cycle.

[13] Chen Y, Gaczynska M, Osmulski P, Polci R, Riley DJ (2010). Phosphorylation by Nek1 regulates opening and closing of voltage dependent anion channel 1. Biochem Biophys Res Commun.

[14] Melo Hanchuk TD, Papa PF, La Guardia PG, Vercesi AE, Kobarg J (2015). Nek5 interacts with mitochondrial proteins and interferes negatively in mitochondrial mediated cell death and respiration. Cell Signal.

[15] Gu Z, Xia J, Xu H, Frech I, Tricot G, Zhan F (2017). NEK2 promotes aerobic glycolysis in multiple myeloma through regulating splicing of pyruvate kinase. J Hematol Oncol.

[16] Basei FL, Meirelles GV, Righetto GL, Dos Santos Migueleti DL, Smetana JHC, Kobarg J (2015). New interaction partners for Nek4.1 and Nek4.2 isoforms: from the DNA damage response to RNA splicing. Proteome Sci.

[17] Qin L, Fan M, Candas D, Jiang G, Papadopoulos S, Tian L (2015). CDK1 enhances mitochondrial bioenergetics for radiation-induced DNA repair. Cell Rep.

[18] Wang Z, Fan M, Candas D, Zhang TQ, Qin L, Eldridge A (2014). Cyclin B1/Cdk1 coordinates mitochondrial respiration for cell-cycle G2/M progression. Dev Cell.

[19] Costa FC, Saito Â, Gonçalves KA, Vidigal PM, Meirelles GV, Bressan GC (2014). Ki-1/57 and CGI-55 ectopic expression impact cellular pathways involved in proliferation and stress response regulation. Biochim Biophys Acta.

[20] Mortz E, Krogh TN, Vorum H, Görg A (2001). Improved silver staining protocols for high sensitivity protein identification using matrix-assisted laser desorption/ionization-time of flight analysis. Proteomics.

[21] Aragão AZB, Belloni M, Simabuco FM, Zanetti MR, Yokoo S, Domingues RR, et al. Novel processed form of syndecan-1 shed from SCC-9 cells plays a role in cell migration. PLoS One. 2012;7(8):e43521. 10.1371/journal.pone.0043521 PMC3419706 22905270

[22] Carazzolle MF, De Carvalho LM, Slepicka HH, Meirelles GV (2014). IIS – integrated Interactome system : a web-based platform for the annotation, analysis and visualization of protein-metabolite-gene-drug interactions by integrating a variety of data sources and tools. PLoS One.

[23] Boyle EI, Weng S, Gollub J, Jin H, Botstein D, Cherry JM (2004). GO::TermFinder - open source software for accessing gene ontology information and finding significantly enriched gene ontology terms associated with a list of genes. Bioinformatics..

[24] Shannon P, Markiel A, Ozier O, Baliga NS, Wang JT, Ramage D (2003). Cytoscape: a software environment for integrated models of biomolecular interaction networks. Genome Res.

[25] Wieckowski MRMR, Giorgi C, Lebiedzinska M, Duszynski J, Pinton P (2009). Isolation of mitochondria-associated membranes and mitochondria from animal tissues and cells. Nat Protoc.

[26] Schindelin J, Arganda-Carreras I, Frise E, Kaynig V, Longair M, Pietzsch T, et al. Fiji: an open-source platform for biological-image analysis. Nat Methods. 2012;7:676–82. 10.1038/nmeth.2019 PMC3855844 22743772

[27] Muñoz JP, Zorzano A. Analysis of mitochondrial morphology and function under conditions of mitofusin 2 deficiency. Mitochondrial Med. 2015;1265:307–20. 10.1007/978-1-4939-2288-8_21 25634283

[28] Melo-Hanchuk TD, Slepicka PF, Pelegrini AL, Menck CFM, Kobarg J (2019). NEK5 interacts with topoisomerase IIβ and is involved in the DNA damage response induced by etoposide. J Cell Biochem.

[29] Bradford MM. A rapid and sensitive method for the quantitation microgram quantities of protein utilizing the principle of protein-dye binding. Anal Biochem. 1976;254:248–54. 10.1016/0003-2697(76)90527-3 942051

[30] Eigentler A, Draxl A, Wiethüchter A, Kuznetsov AV (2015). Laboratory protocol: citrate synthase a mitochondrial marker enzyme. Mitochondrial Physiol Netw.

[31] Vuzman D, Hoffman Y, Levy Y. Modulating protein-DNA interactions by post-translational modifications at disordered regions. Pac Symp Biocomput. 2012:188–99. PMID:22174274. 22174274

[32] Coene KLM, Mans DA, Boldt K, Gloeckner CJ, Van J, Bolat E, et al. The ciliopathy-associated protein homologs RPGRIP1 and RPGRIP1L are linked to cilium integrity through interaction with Nek4 serine / threonine kinase. Hum Mol Genet. 2011;20:3592–605. 10.1093/hmg/ddr280 21685204

[33] De Souza EE, Meirelles GV, Godoy BB, Perez AM, Smetana JHC, Doxsey SJ (2014). Characterization of the human NEK7 interactome suggests catalytic and regulatory properties distinct from those of NEK6. J Proteome Res.

[34] Hecht SM. Bleomycin: new perspectives on the mechanism of action. J Nat Prod. 2000;63(1):158–68. 10.1021/np990549f 10650103

[35] NCBI National Center for Biotechnology Information (2017). Bethesda Natl. Libr. Med.

[36] The Universal Protein Resource (UniProt). Nucleic Acids Res. 2007;36:D190–5. 10.1093/nar/gkm895 PMC2238893 18045787

[37] Jastroch M, Divakaruni AS, Mookerjee S, Treberg JR, Brand MD (2010). Mitochondrial proton and electron leaks. Essays Biochem.

[38] Hoerter J, Gonzalez-Barroso MDM, Couplan E, Mateo P, Gelly C, Cassard-Doulcier AM (2004). Mitochondrial uncoupling protein 1 expressed in the heart of transgenic mice protects against ischemic-reperfusion damage. Circulation.

[39] Ruprich-Robert G, Zickler D, Berteaux-Lecellier V, Vélot C, Picard M (2002). Lack of mitochondrial citrate synthase discloses a new meiotic checkpoint in a strict aerobe. EMBO J.

[40] Hwang J, Kim YY, Huh S, Shim J, Park C, Kimm K (2005). The time-dependent serial gene response to zeocin treatment involves caspase-dependent apoptosis in HeLa cells. Microbiol Immunol.

[41] Plaitakis A, Kalef-Ezra E, Kotzamani D, Zaganas I, Spanaki C. The glutamate dehydrogenase pathway and its roles in cell and tissue biology in health and disease. Biology. 2017;8(6):E11. 10.3390/biology6010011 PMC5372004 28208702

[42] Tilokani L, Nagashima S, Paupe V, Prudent J. Mitochondrial dynamics: overview of molecular mechanisms. Essays Biochem. 2018;62:341–60. 10.1042/EBC20170104 PMC6056715 30030364

[43] Canugovi C, Maynard S, Bayne AC, Sykora P, Tian J, de Souza-Pinto NC (2010). The mitochondrial transcription factor a functions in mitochondrial base excision repair. DNA Repair.

[44] Ekstrand MI, Falkenberg M, Rantanen A, Park CB, Gaspari M, Hultenby K, et al. Mitochondrial transcription factor a regulates mtDNA copy number in mammals. Hum Mol Genet. 2004;13:935–44. 10.1093/hmg/ddh109 15016765

[45] Szabó I, Zoratti M (1993). The mitochondrial permeability transition pore may comprise VDAC molecules. I. Binary structure and voltage dependence of the pore. FEBS Lett.

[46] Kluckova K, Bezawork-Geleta A, Rohlena J, Dong L, Neuzil J. Mitochondrial complex II, a novel target for anti-cancer agents. Biochim Biophys Acta Bioenerg. 2013;1827(5):552–64. 10.1016/j.bbabio.2012.10.015 23142170

[47] Desler C, Hansen TL, Frederiksen JB, Marcker ML, Singh KK, Juel RL. Is there a link between mitochondrial reserve respiratory capacity and aging? J Aging Res. 2012;2012:192503. 10.1155/2012/192503 PMC3375017 22720157

[48] Divakaruni AS, Brand MD. The regulation and physiology of mitochondrial proton leak. Physiology. 2011;26:192–205. 10.1152/physiol.00046.2010 21670165

[49] Lin CC, Cheng TL, Tsai WH, Tsai HJ, Hu KH, Chang HC, et al. Loss of the respiratory enzyme citrate synthase directly links the Warburg effect to tumor malignancy. Sci Rep. 2012;2:785. 10.1038/srep00785 PMC3492867 23139858

[50] Yan C, Duanmu X, Zeng L, Liu B, Song Z. Mitochondrial DNA: Distribution, Mutations, and Elimination. Cells. 2019;8(4):379. 10.3390/cells8040379 PMC6523345 31027297

[51] Kawamura K, Qi F, Kobayashi J. Potential relationship between the biological effects of low-dose irradiation and mitochondrial ROS production. J Radiat Res. 2018:59:ii91–7. 10.1093/jrr/rrx091 PMC5941154 29415254

[52] Zhang J, Li D, Qu F, Chen Y, Li G, Jiang H, et al. Association of leukocyte mitochondrial DNA content with glioma risk: evidence from a Chinese case-control study. BMC Cancer. 2014;14:680. 10.1186/1471-2407-14-680 PMC4177174 25234800

[53] Shen J, Platek M, Mahasneh A, Ambrosone CB, Zhao H (2010). Mitochondrial copy number and risk of breast cancer: a pilot study. Mitochondrion..

[54] Wang Y, Liu VWS, Xue WC, Tsang PCK, Cheung ANY, Ngan HYS (2005). The increase of mitochondrial DNA content in endometrial adenocarcinoma cells: a quantitative study using laser-captured microdissected tissues. Gynecol Oncol.

[55] Ryzhkova AI, Sazonova MA, Sinyov VV, Galitsyna EV, Chicheva MM, Melnichenko AA, et al. Mitochondrial diseases caused by mtDNA mutations: a mini-review. Clin Risk Manag. 2018;14:1933–42. 10.2147/TCRM.S154863 PMC6186303 30349272

[56] Rose S, Frye RE, Slattery J, Wynne R, Tippett M, Pavliv O, et al. Oxidative stress induces mitochondrial dysfunction in a subset of autism lymphoblastoid cell lines in a well-matched case control cohort. PLoS One. 2014;9(1):e85436. 10.1371/journal.pone.0085436 PMC3885720 24416410

