# Supplementary material for: NEK10 interactome and depletion reveal new roles in mitochondria
Source: Proteome Sci. 2020 Apr 28;18:4. doi: 10.1186/s12953-020-00160-w (PMC7189645; doi:10.1186/s12953-020-00160-w)
Supplement: Supplementary file 1 — Additional file 1: Figure S1. NEK10 interacts with mitochondrial partners. Interaction network of human NEK10 with proteins partners, identified by IP-LC-MS/MS. Tryptic-digested peptides from FLAG or FLAG-NEK10 immunoprecipitates were analyzed by mass spectrometry and protein partners were identified. The samples were untreated (A) or treated with zeocin (B). The proteomic data retrieved from IP-LC-MS/MS was submitted to the Integrated Interactome System (IIS) platform (National Laboratory of Biosciences, Campinas, Brazil) [Carazzolle et al., 2014] [22]. The protein-protein interaction network (PPI) was generated using Cytoscape software [Shannon et al., 2003] [24]. Figure S2. Mitochondrial fractionation. Mitochondria from A- MRC5 cells and B- HEK293T cells were isolated and the localization of NEK10 was analyzed by Western blot using anti-NEK10 antibody. Postnuclear supernatant (PNS), cytosol (CYT) and mitochondrial (MITO) fractions were analysed with anti-Lamin A/C, anti- GAPDH, anti-Tubulin A, anti-OXPHOS and anti-VDAC, to access fractionation purity. The letter A and the red arrows indicate the 133 kDa isoform present in PNS and CYTO. The letter B and the blue arrows indicate the 80 kDa isoform present in the mitochondrial fraction (MITO). Figure S3. Validation of NEK10 depletion in HeLa cells by shRNA. Two different pLKO-shRNAs were designed to target NEK10 (shNEK10–89 and shNEK10–90, named here as sh89 and sh90, respectively). A- Immunoblotting of HeLa pLKO, HeLa pLKO-sh89 and HeLa pLKO-sh90 cells lysates with anti-NEK10 antibody and anti-GAPDH antibody. B and C- The graphs B and C show the percentage of 133 kDa and 80 kDa NEK10 depletion, respectively. The quantification is shown from n = 5 independent experiments. The letter A and red arrows indicate 133 kDa NEK10 isoform. The letter B and blue arrows indicate 80 kDa NEK10 isoform. Figure S4. NEK10 antibody presented specificity. HeLa cells transfected with FLAG-Nek10 presented increased fluorescence intensi [file 12953_2020_160_MOESM1_ESM.pptx]

## Slide 1
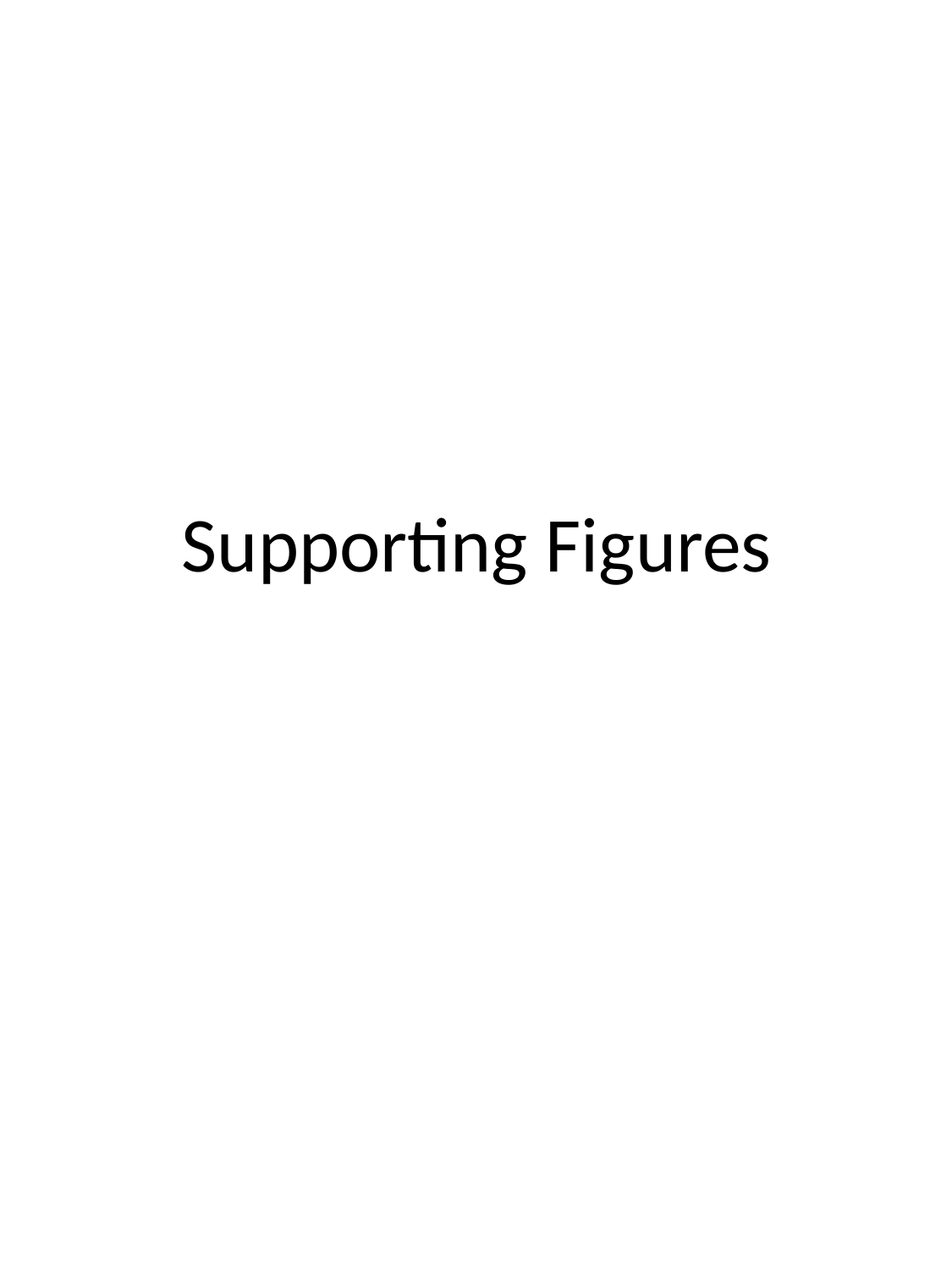

# Supporting Figures

## Slide 2
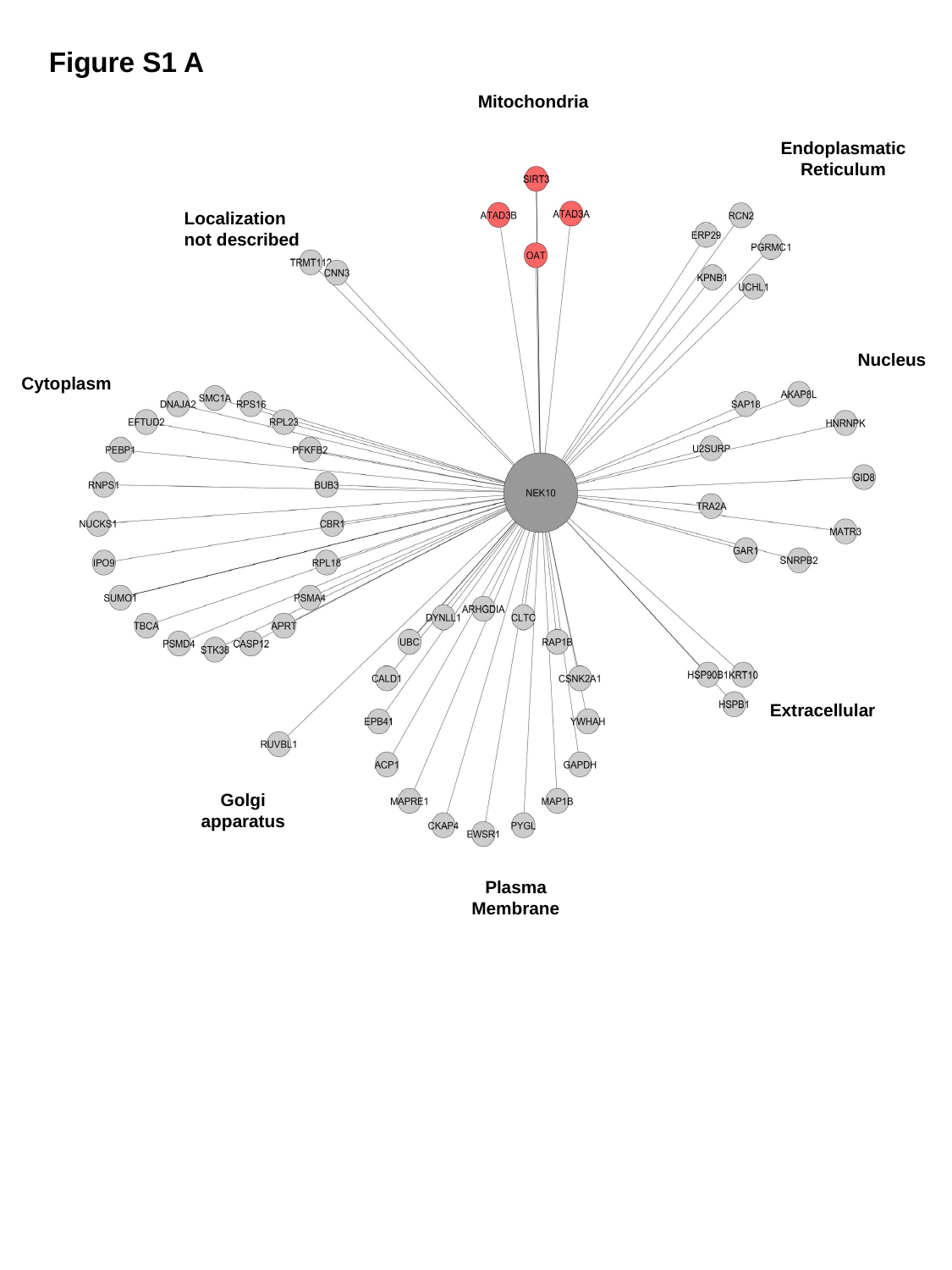

Figure S1 A
Mitochondria
Endoplasmatic Reticulum
Localization not described
Nucleus
Cytoplasm
Extracellular
Golgi apparatus
Plasma Membrane

## Slide 3
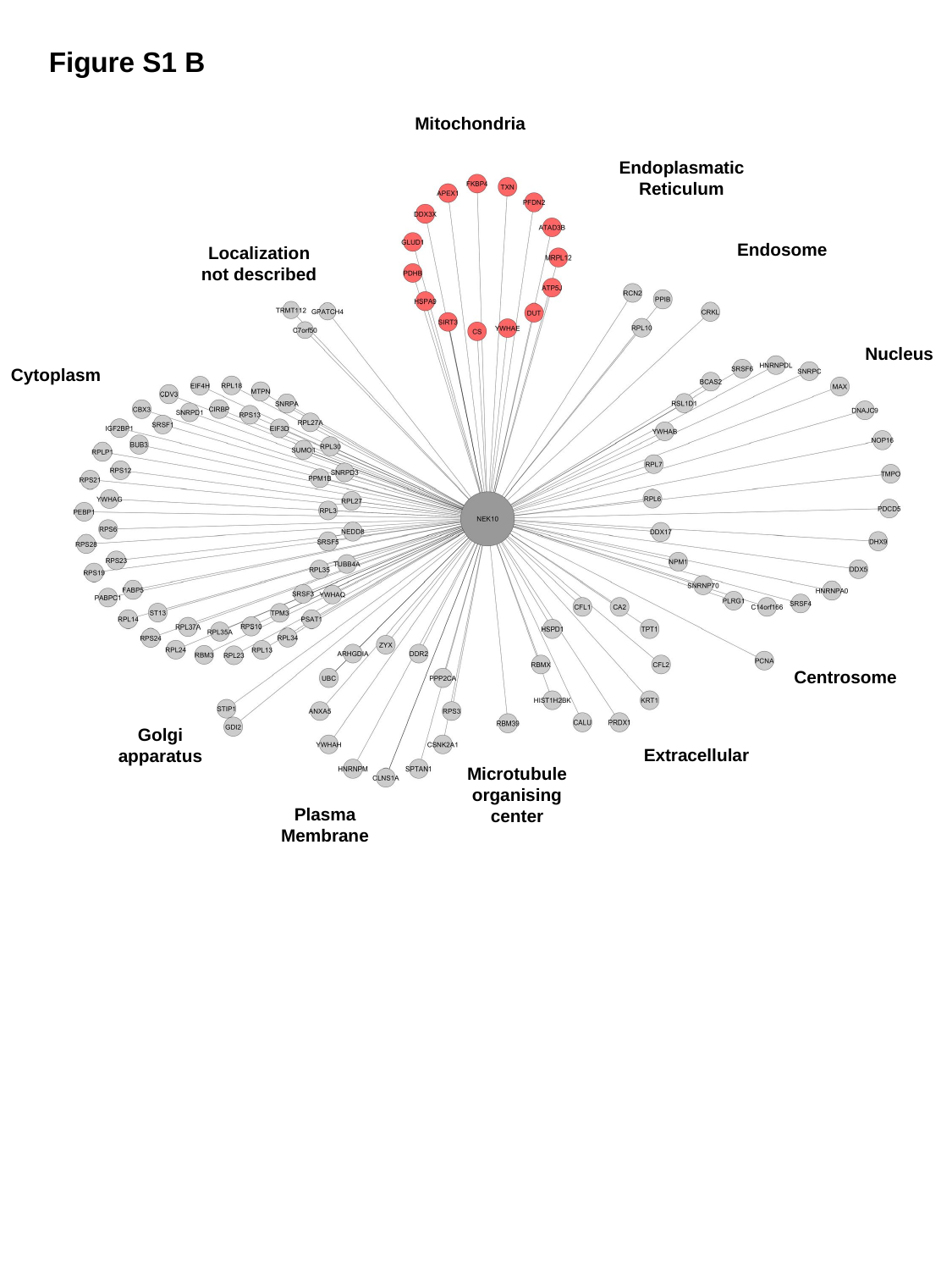

Figure S1 B
Mitochondria
Endoplasmatic Reticulum
Endosome
Localization not described
Nucleus
Cytoplasm
Centrosome
Golgi apparatus
Extracellular
Microtubule organising center
Plasma Membrane

## Slide 4
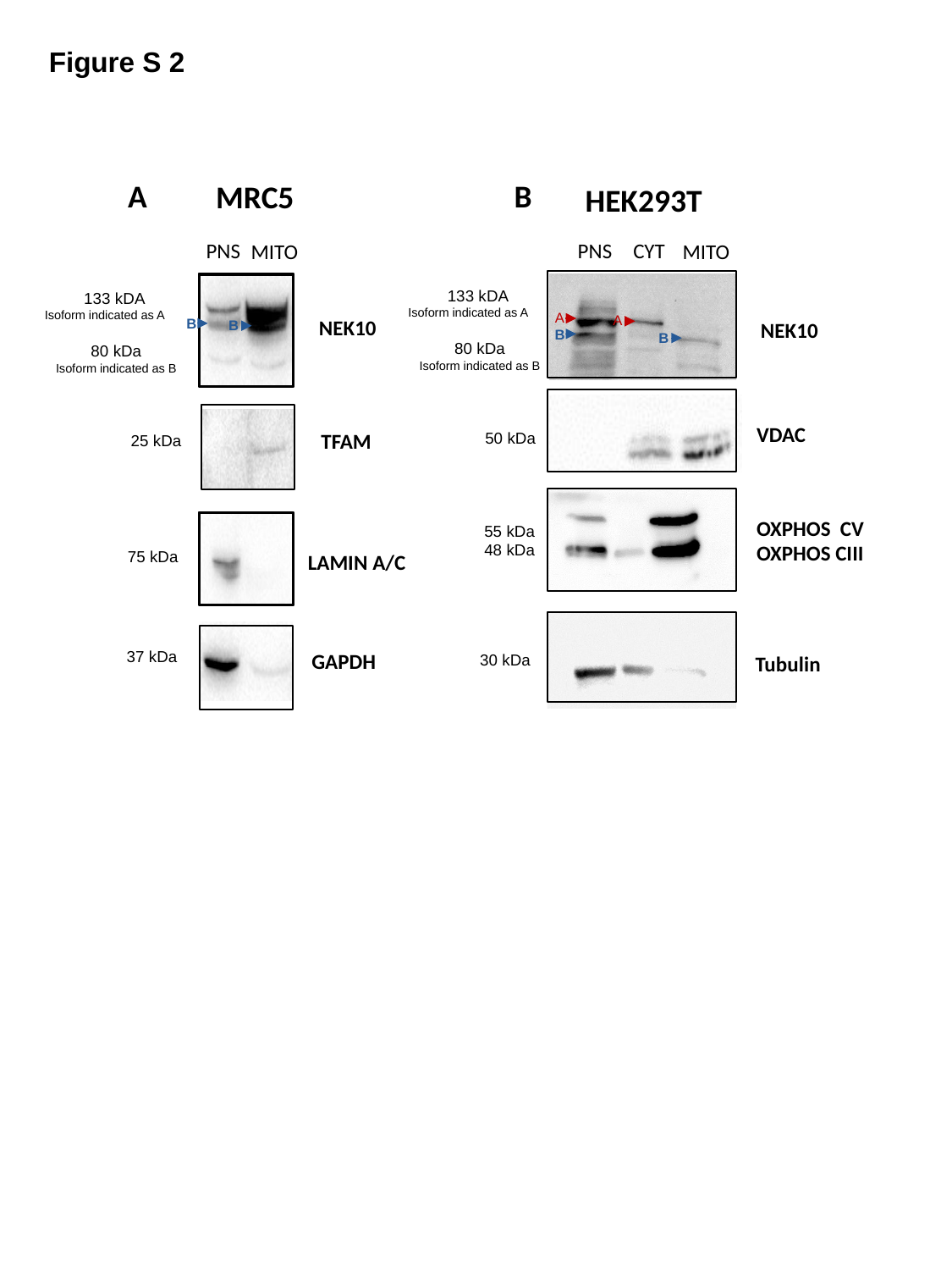

Figure S 2
A
B
MRC5
HEK293T
CYT
PNS
MITO
PNS
MITO
NEK10
NEK10
VDAC
TFAM
50 kDa
25 kDa
OXPHOS CV
OXPHOS CIII
55 kDa
48 kDa
75 kDa
LAMIN A/C
37 kDa
GAPDH
30 kDa
Tubulin
133 kDA
Isoform indicated as A
80 kDa
Isoform indicated as B
133 kDA
Isoform indicated as A
80 kDa
Isoform indicated as B
A
A
B
B
B
B

## Slide 5
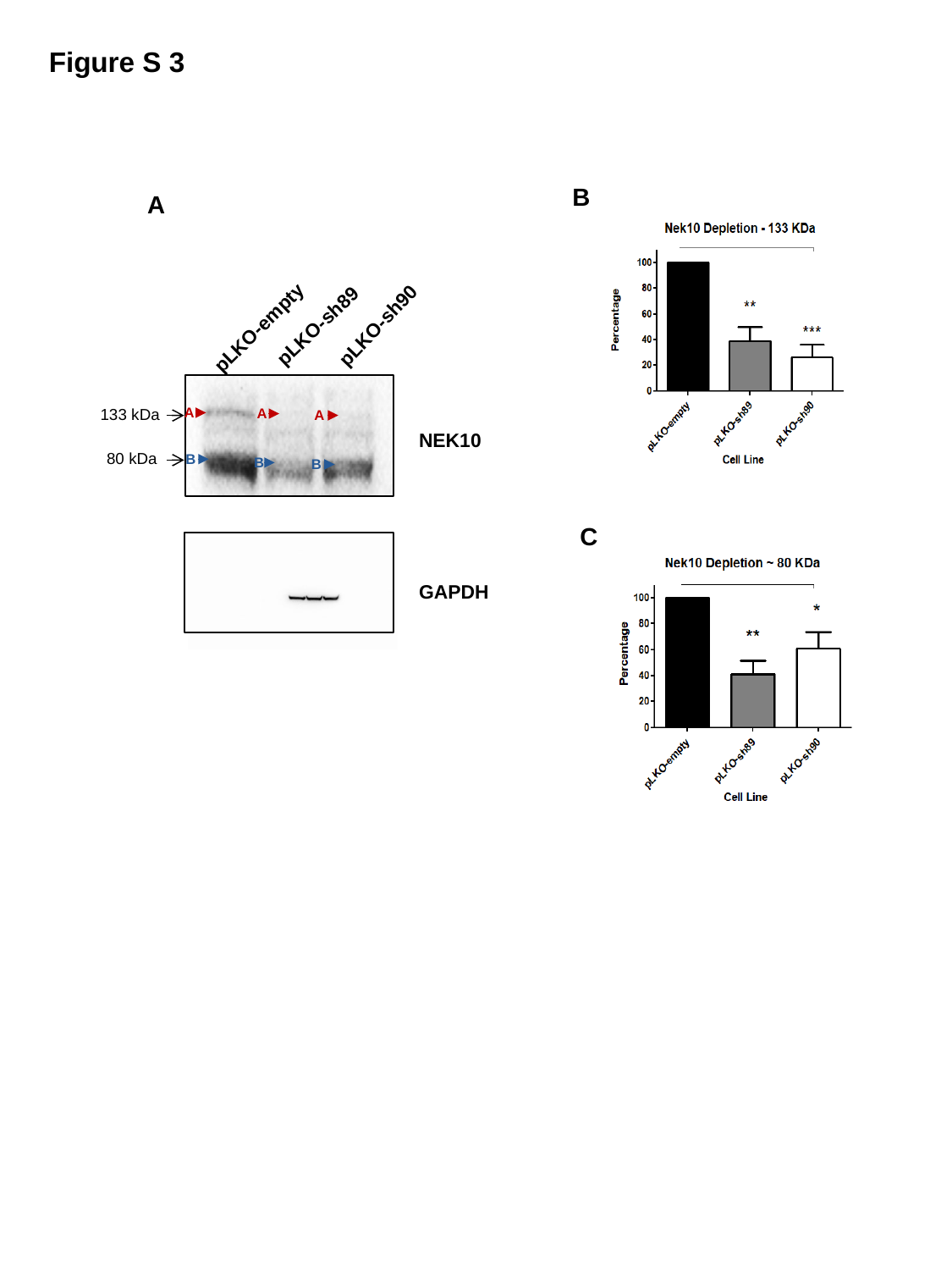

Figure S 3
B
A
pLKO-sh90
pLKO-sh89
pLKO-empty
133 kDa
NEK10
80 kDa
GAPDH
C
A
A
A
B
B
B

## Slide 6
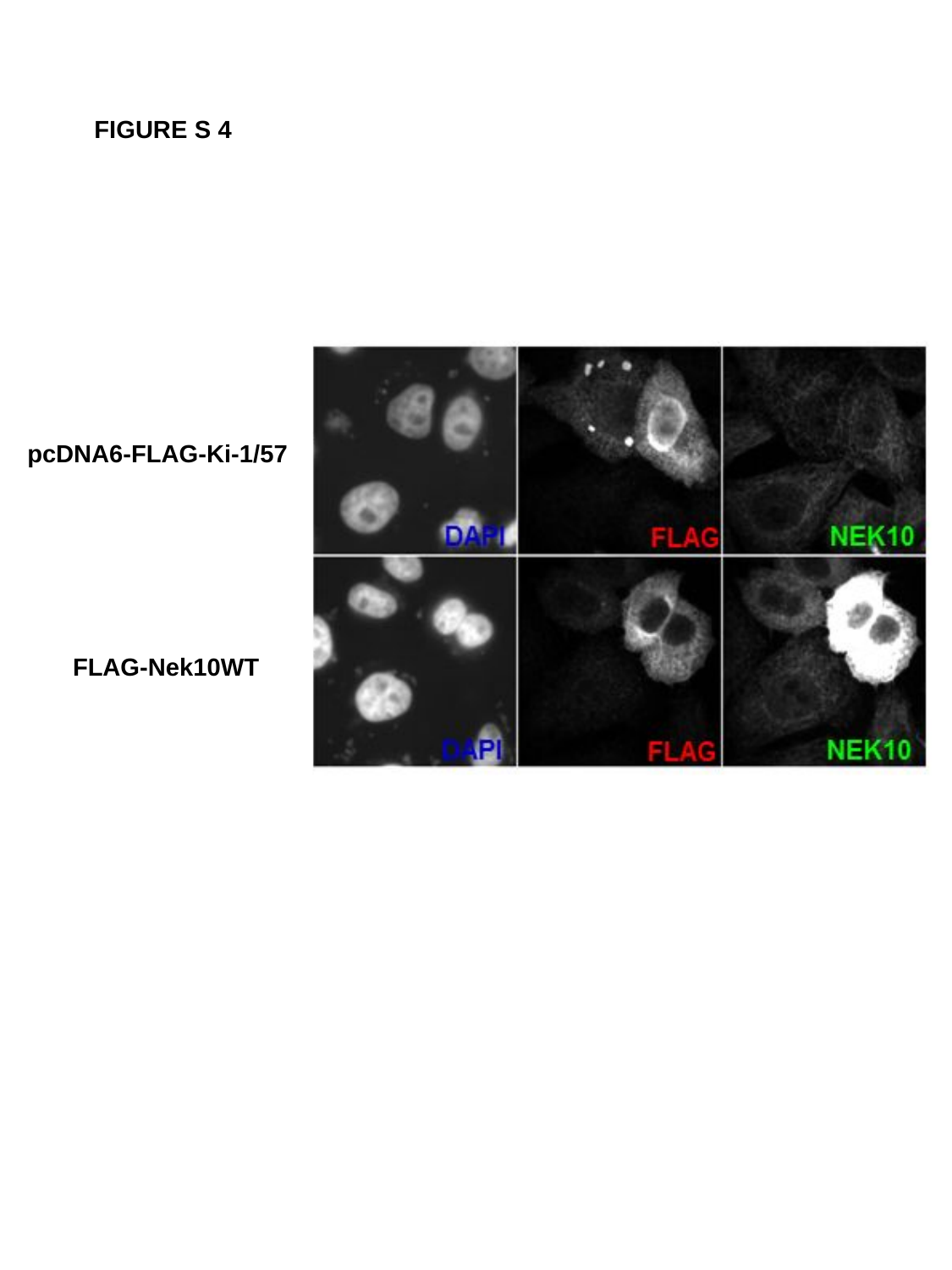

FIGURE S 4
FLAG-Nek10WT
pcDNA6-FLAG-Ki-1/57

## Slide 7
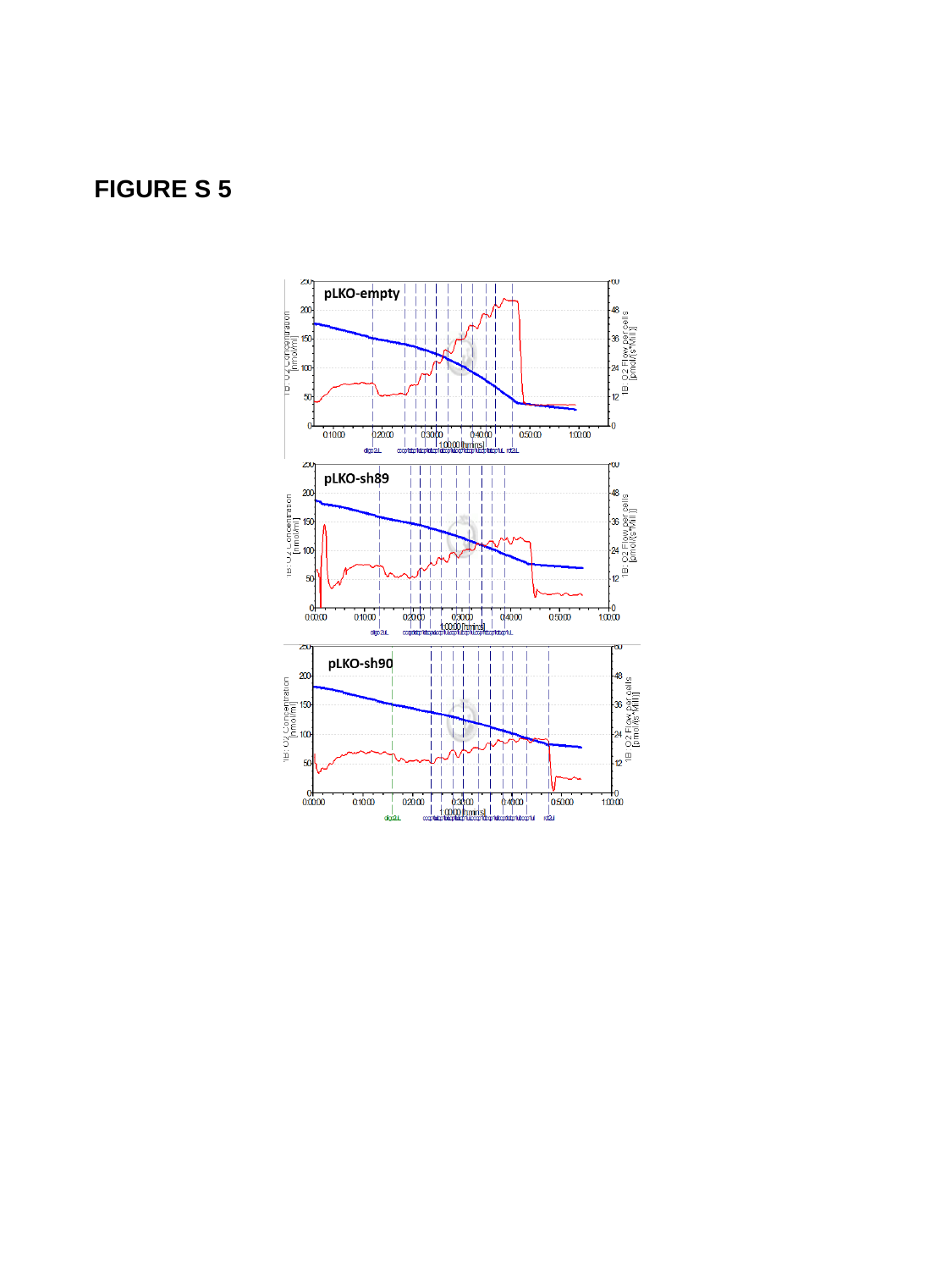

FIGURE S 5
